# Conserved loci of leaf and stem rust fungi of wheat share synteny interrupted by lineage-specific influx of repeat elements

**DOI:** 10.1186/1471-2164-14-60

**Published:** 2013-01-29

**Authors:** John P Fellers, Bahram M Soltani, Myron Bruce, Rob Linning, Christina A Cuomo, Les J Szabo, Guus Bakkeren

**Affiliations:** 1 USDA-ARS, Hard Winter Wheat Genetics Research Unit, Department of Plant Pathology, Manhattan, KS, 66506, USA; 2 Agriculture & Agri-Food Canada, Pacific Agri-Food Research Centre, Summerland, BC, V0H 1Z0, Canada; 3 Broad Institute, 7 Cambridge Center, Cambridge, MA, 02142, UK; 4 USDA-ARS, Cereal Disease Laboratory, 1551 Lindig, St. Paul, MN, 55108, UK

**Keywords:** Wheat leaf rust, BAC construction, Synteny

## Abstract

**Background:**

Wheat leaf rust (*Puccinia triticina* Eriks; *Pt*) and stem rust fungi (*P. graminis f.sp. tritici; Pgt*) are significant economic pathogens having similar host ranges and life cycles, but different alternate hosts. The *Pt* genome, currently estimated at 135 Mb, is significantly larger than *Pgt*, at 88 Mb, but the reason for the expansion is unknown. Three genomic loci of *Pt* conserved proteins were characterized to gain insight into gene content, genome complexity and expansion.

**Results:**

A bacterial artificial chromosome (BAC) library was made from *P. triticina* race 1, BBBD and probed with *Pt* homologs of genes encoding two predicted *Pgt* secreted effectors and a DNA marker mapping to a region of avirulence. Three BACs, 103 Kb, 112 Kb, and 166 Kb, were sequenced, assembled, and open reading frames were identified. Orthologous genes were identified in *Pgt* and local conservation of gene order (microsynteny) was observed. Pairwise protein identities ranged from 26 to 99%. One *Pt* BAC, containing a RAD18 ortholog, shares syntenic regions with two *Pgt* scaffolds, which could represent both haplotypes of *Pgt*. Gene sequence is diverged between the species as well as within the two haplotypes. In all three BAC clones, gene order is locally conserved, however, gene shuffling has occurred relative to *Pgt*. These regions are further diverged by differing insertion loci of LTR-retrotransposon, *Gypsy*, *Copia*, *Mutator*, and *Harbinger* mobile elements. Uncharacterized *Pt* open reading frames were also found; these proteins are high in lysine and similar to multiple proteins in *Pgt*.

**Conclusions:**

The three *Pt* loci are conserved in gene order, with a range of gene sequence divergence. Conservation of predicted haustoria expressed secreted protein genes between *Pt* and *Pgt* is extended to the more distant poplar rust, *Melampsora larici-populina*. The loci also reveal that genome expansion in *Pt* is in part due to higher occurrence of repeat-elements in this species.

## Background

Plants and pathogens are in a constant struggle as each co-evolves to adapt to genomic changes. Plant genomes are adapting to different modes of infection by pathogens while pathogens are evolving different avenues to circumvent defense systems of their respective hosts. Rust fungi are among the most economically important pathogens, yet are part of elusive host-pathogen systems. The order Pucciniales (formerly Uredinales or Urediniomycetes) contains over 7,000 different species from 100 genera [1]. Adding to the complexity, individual cereal crops can be infected by several rust fungi adapted to the specific crop.

Cereal rust fungi are obligate biotrophs and have alternate hosts where sexual recombination takes place, allowing for diversification of the population [2]. The life cycle of cereal rust fungi begins with a urediniospore landing on a leaf surface and germinating in the presence of adequate humidity. A germtube emerges and moves towards a stomate via a thigmotrophic response and probable chemical clues [3] where an appressorium will form. A hypha grows inside the substomatal space until a mesophyll cell is encountered. The fungus will penetrate the cell wall and produce a haustorium by invagination of the plasma membrane [4,5] At each stage of infection, the fungus is postulated to secrete effectors to inhibit cell defenses and reprogram cells to redirect nutrients. Though some candidate effectors are shared among the rust fungi, most are specific to their host and include transcription factors, zinc finger proteins, small secreted proteins and cysteine-rich proteins [6]. Certain classes of effectors, such as ones modulating host immunity, are believed to rapidly change to overcome resistance, however, the mechanisms generating this variation are not known. In several studied pathogens, certain classes of predicted effectors are found in variable and highly mutagable regions of the genome. Mobile elements induced mutations in effectors in *Phytophthora*[7], *Magnaporthe*[8], and *Leptosphaeria*[9] while *Fusarium oxysporum* has a specialized chromosome with effectors [10,11]. Effectors can be clustered in the genome (*Ustilago;*[12]) including at telomeres (*Fusarium,*[13]; *Magnaporthe;*[14]). Avirulence genes from the flax rust fungus, *Melampsora lini* are all small secreted proteins [15,16]. Currently, two effectors have been identified in urediniospores of *Puccinia graminis f.sp. tritici* (*Pgt*) that induce the *in vivo* phosphorylation and degradation of the barley resistance protein, RPG1 [17].

Sequencing technology has made significant advancements in recent years. Complete genomes of more species, including fungi, are being sequenced. Comprehensive catalogs of genes can be generated, annotated, and comparisons made to other genomes. Core sets of genes needed for function, adaptations for life cycle, and host specificity can now be found. Comparisons of several obligate fungal plant parasites have identified common losses of genes involved in nitrate and sulfur metabolism [6,18]. *Melampsora larici-populina* (*Mlp*) and *Pgt* have approximately 8,000 orthologous genes which could be suggested as a core set needed for biotrophism. However, 74% and 84% of the secreted proteins, respectively, are lineage specific [6] suggesting proteins that are needed for the individual life cycle. Corn pathogens, *U. maydis* and *S. reilianum* are also closely related and share 71% of effector genes in so-called divergence clusters. However, 10% are *U. maydis* specific while 19% are specific to *S. reilianum*[19].

*Puccinia triticina* (*Pt*) is the causal agent of wheat leaf rust and new races emerge each year aided by a crop monoculture placing a strong selection pressure on the pathogen. Genetic variation is generally believed to increase through sexual recombination to generate new allele combinations. Two related wheat rust fungi, *Pgt* and *P. striiformis* f.sp. *tritici* (*Pst*), causing stripe rust, have a sexual cycle on North American *Berberis spp*. and have a greater race variability where the alternate host is present [20]. The *Pt* aeciospore stage is on *Thalictrum spp.* and *Isopyrum*, found mainly in the Mediterranean region, however, other *Thalictrum* species present in North America can support a reduced level of infection [21] but are generally resistant to *Pt*[22,23]. Populations are essentially asexual; supported by the lack of recombination found in numerous North American races [24-28]. A parasexual cycle may exist allowing recombination since germtube fusion, nuclear migration, and bridging structures between nuclei have been observed in *Pt*[29].

The obligate biotrophic nature of cereal rusts makes experimental manipulation difficult, however, genomics provides a means of studying evolution and gene function. We set out to understand the genome variation of two rust fungi at three regions. A *Pt* bacterial artificial chromosome (BAC) library was made and clones were identified using three probes that would isolate regions of predicted secreted proteins and avirulence. Sequenced DNA regions of *Pt* were compared to syntenic regions in two rust species with complete genome sequences, *Pgt, and Mlp*[6], and evaluated for genomic conservation, expansion and mutations.

## Results

### BAC library construction

Urediniospores harbor two haploid nuclei with an estimated total genome complexity for *Pt* of approximately 135 Mb, based on comparative DNA fluorescence (L. Szabo, unpublished) and the current total size of the genome assembly (http://www.broadinstitute.org/annotation/genome/puccinia_group/GenomeStats.html). The generated *P. triticina* BAC library contained 15,360 clones arrayed in 384 well plates with an average insert size of 105 kb representing an estimated 10 to 12 genome equivalents. A single-copy probe identified nine positive clones on high density filters, and assuming fragments were randomly cloned during library construction, this is in agreement with the estimated genome coverage.

### BAC clone selection, sequencing and characterization

Three genomic regions were targeted for comparison. Previous work had mapped a *Pgt* RAD18 homolog in a genomic region harboring an avirulence gene [30]. Using PgtRAD18 as a reference, PT0313.J16.C21 (GenBank accession number GR497566) was identified from a *Pt* EST database using TBLASTN (E value = e-107; [31,32]) and used as a probe. Nine positive BAC clones were found and clone 1F16 (Pt1F16) was selected for sequencing because of its longer length and the centralized location of PtRAD18 within the BAC clone. Sequences from Pt1F16 were assembled into two contiguous sequences of 39,219 and 63,874 bp, totaling 103,093 bp (GenBank JX489506). The GC content of these sequences was 47%. Subclones were generated spanning the gap for orientating and ordering of the two contigs. However, due to a region of 60 near-perfect 46 bp repeats of ACCAGCCCGCCGAGAGGAAGCCCTCTCGGCGAGCTGGTGTGTGTAT, the gap could not be closed. FGENESH, with gene models from the *Puccinia* group genome project (http://www.broadinstitute.org/annotation/genome/puccinia_group/), predicted 30 open reading frames (ORFs) ranging from 210 to 4,077 bp in length.

In a functional screen of *Uromyces viciae-fabae*, secreted peptide effector protein UF5 was related to the flax rust *Melampsora lini* haustorial-expressed secreted protein HESP-379 [16]. A *Pgt* genome search revealed several predicted secreted protein homologs in close proximity, suggesting the presence of small clusters of predicted secreted proteins. UF5 (Genbank ES608162) aligned with two predicted secreted proteins, PGTG_03708 (E score 5 e^-33^) and PGTG_03709 (E score 0), both transcribed and located 513 bp apart on the *Pgt* contig. Using these *Pgt* sequences, PtContig18 (Genbank accession HP451841) and PtContig7347 (Genbank accession HP458556) were identified by a BLASTN *Pt* EST database search. A PCR product from the cDNA clone, *Pt* EST PT0061b.D10.TB that aligned to Contig18 (GenBank accession EC400508), was used as a probe to identify *Pt* BAC PtHSP02. Sequencing of this BAC resulted in four assembled contigs. Gaps could be spanned and thus the contigs could be ordered and oriented. Sizes of the contigs in bp were 16,991, 30,055, 5,014, and 60,277 for a total of 112,337 bp (GenBank JX489507). Gaps were present in regions of repeated DNA and could not be assembled. GC content was 46.3% and FGENESH predicted 31 ORFs in the contig ranging from 174 bp to 7,167 bp in length. The smaller ORFs were generally within repeated elements.

The bean rust effector UfHSP42c Uf011 (GenBank ES608167; [33]) matched three predicted protein sequences in *Pgt*, PGTG_17547 (E Score 0), PGTG_17548 (E Score 1 e^-21^) and PGTG_17549 (E Score 1 e^-4^). UfHSP42c matched five *Pt* ESTs, including clone PT0131d.B10.BR (GenBank accession EC414978) from which probes were derived to identify *Pt* BAC clone HSP04. Sequencing of HSP04 produced two contiguous sequences of 9,276 bp and 157,027 bp for a total of 166,303 bp (GenBank JX489508). GC content was 46.3% and 61 ORFs were predicted ranging from 120 bp to 5,214 bp in length.

### BAC annotation

The predicted ORFs from each BAC clone were aligned using BLASTN to the *Pgt* genome, *Pgt* predicted transcripts and *Pt* ESTs, and using BLASTX, to the *Pgt, Mlp,* and *U. maydis (Um)* predicted proteomes (Table 1). Pt1F16 had nine ORFs with synteny in *Pgt.* Identity across the protein sequences ranged from 37-87% in these alignments and putative annotations could be assigned to five of the proteins. Pt1F16-4 contained many gaps when compared to PGTG_13013. Proteins Pt1F16-5, 6, 7, 8 and 9 aligned with two proteins each from *Pgt*. Pt1F16-7 aligned with PgtRAD18, which has one copy in each of the *Pgt* haplotype genomes. All but one homolog could also be found in *Mlp* and four were represented in *Um* (Table 1).

**Table 1:** **Gene features in three *Puccinia triticina *BAC clones and their alignment to other sequenced Basidiomycetes, *P. graminis tritici (Pgt), Melampsora larici-populina (Mlp), *and *Ustilago maydis (Um)***

***P. triticina***	*Pgt*Gencxxe Feature*	***Pgt protein***	***Mlp***	***Um***	**BLASTX**
**ORF**		**identities**	gene feature**	**gene feature**	**Annotation**
1 F16-1	PGTG_12990	54%	35.90208/11	-	Similar to Uhrf1
1 F16-2	PGTG_13012	60%	104.11397	Um_00786	Hypothetical protein
1 F16-3	PGTG_13013	63%	74. 94858	-	Similar to esterase
1 F16-4	PGTG_13016	37%	-	-	Predicted protein
	PGTG_18731	64%			
1 F16-5	PGTG_13018	67%	49.39112	Um_02725	Similar to molybdopterin synthase sulpherylase
	PGTG_18732	68%			
1 F16-6	PGTG_13021	68%	74.73436	-	Hypothetical protein
	PGTG_18735	64%			
1 F16-7	PGTG_13023	56%	74.94864	Um_05085	RAD18
	PGTG_18741	56%			
1 F16-8	PGTG_13024	64%	74.000024	-	cystein rich SCP-like extracellular protein
	PGTG_18744	65%			
1 F16-9	PGTG_13026	87%	74.50754/28052	Um_00594	Similar to pyruvate dehydrogenase complex
	PGTG_18746	86%			
HSP02-1	PGTG_3730/1	79%	22.87674	Um_00736	Conserved protein
HSP02-2	PGTG_6672	48%	68.40205	Um_04270	Aspartyl-tRNA synthetase
HSP02-3	PGTG_3706	30%	-	-	-
HSP02-4	PGTG_3708	69%	2.70587	Um_01555	*Mlp* HESP-379
HSP02-5	PGTG_3709	83%	2.70587	Um_01555	*Mlp* HESP-379
HSP02-6	PGTG_3727	100%	2.76428	Um_00703	G-protein beta subunit
HSP02-7	PGTG_3728	68%	2.115002	Um_03486	Nucleotide-binding protein 2
HSP02-8	PGTG_3729	82%	2.46419	Um_05743	Pre-mRNA splicing factor ATP-dependent RNA helicase PRP16
HSP02-9	PGTG_3730	87%	10.115914	Um_04551	Similar to cyclin *Ctk2*
HSP04-1	PGTG_16978	36%	-	-	Predicted protein
HSP04-2	PGTG_16976	73%	27.88522	Um_00639	Nucleoporin-like
HSP04-3	PGTG_10949	95%	47.72927	Um_02479	60S ribosomal protein
HSP04-4	PGTG_02586	33%	27.88520	-	Heat shock protein 90
HSP04-5	PGTG_14539	41%	-	-	Predicted protein
HSP04-6	PGTG_17549	26%	-	-	Predicted secreted protein
HSP04-7	PGTG_17549	60%	-	-	Predicted secreted protein
HSP04-8	PGTG_17547/8	76%	16.85997	-	*Uf*-HSP42c
HSP04-9	PGTG_17547/8	71%	16.85997	-	*Uf*-HSP42c
HSP04-10	PGTG_05205	82%	22.48630	-	Integral membrane protein
HSP04-11	PGTG_17545	52%	-	Um_00662	Predicted protein
HSP04-12	PGTG_17544	83%	23.87824	Um_03820	Vacuolar sorting protein PEP5
HSP04-13	PGTG_17543	62%	23.72100	Um_02189	Hypothetical protein
HSP04-14	PGTG_17537/8	38%	-	-	Predicted protein

**Figure 1:**
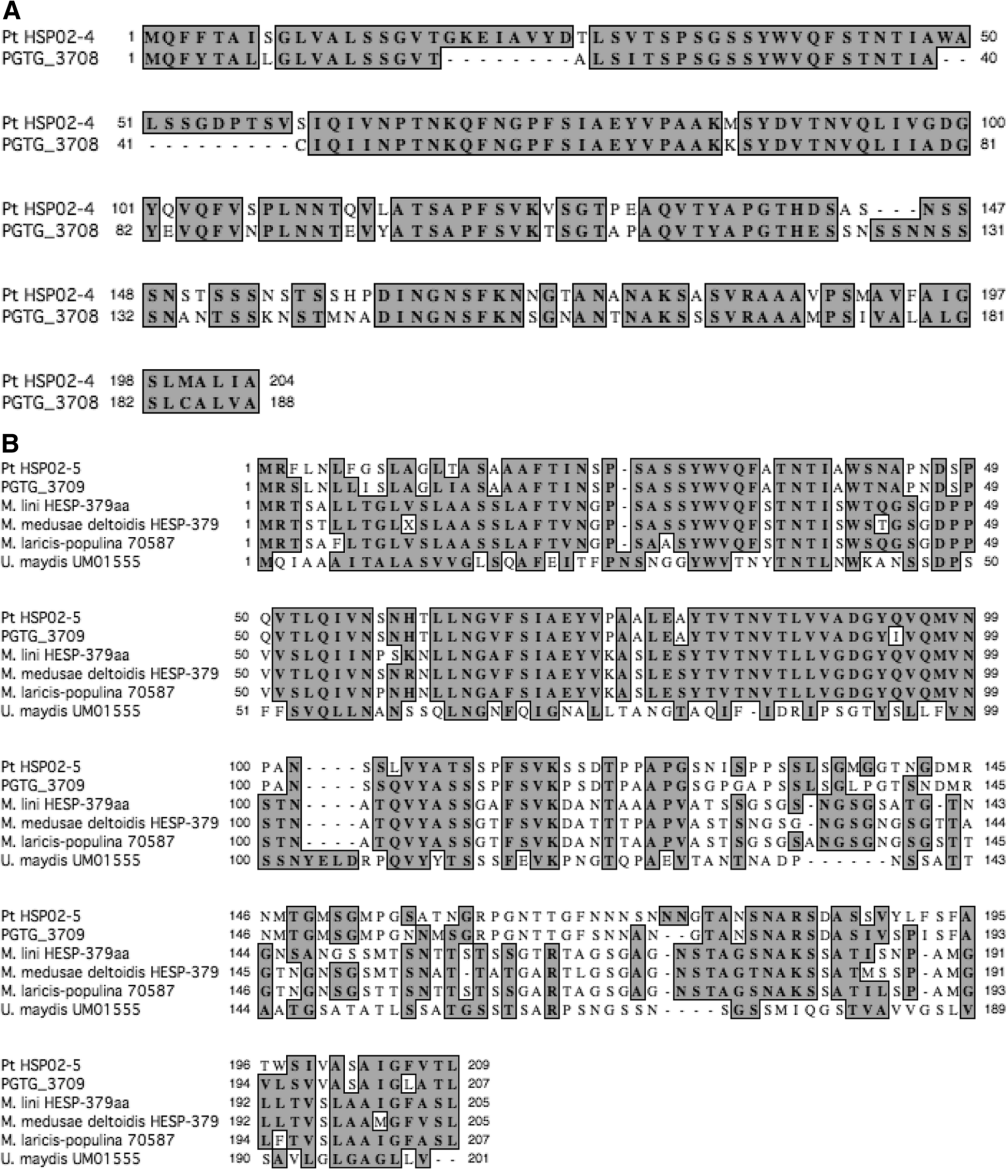
**ClustalW alignments of secreted proteins coded on BAC PtHSP02. A**) PtHSP02-4 aligned to a *Pgt* homolog. **B**) PtHSP02-5 aligned to homologs from *Pgt, Melampsora lini, M. medusae deltoidis, Mlp* and *U. maydis*.

* *Pgt* gene features indicate gene nUmber assigned by the Broad institute during assembly (http://www.broadinstitute.org/annotation/genome/puccinia_group/ verified September 28, 2012). Two nUmbers indicate different scaffolds, as highlighted in Figure 1.

** *Mlp* gene features are indicated by scaffold nUmber; gene nUmber as assigned by the Joint Genome Initiative at the Department of Energy (http://genomeportal.jgi-psf.org/Mellp1/Mellp1.home.html verified September 28, 2012).

Nine predicted proteins in PtHSP02 were confirmed through EST sequence alignment [32] and a putative function could be assigned to eight of them. Alignment identity ranged from 30-100% in PtHSP02. Eight homologs could be found in both *Mlp* and *Um* in PtHSP02. The most highly conserved protein is PtHSP02-6, a G-protein ß-subunit containing a conserved WD-40 repeat motif. The first 343 amino acids were 100% identical to PGTG_03727 and 99% to *Mlp* accession GL883091 (Table 1). Conversely, PtHSP02-3 was only 30% identical to PGTG_3706 and had no homologs in the other two fungi. PtHSP02-4 and PtHSP02-5 aligned with *Mlp* HESP-379, the haustorial expressed predicted secreted protein homolog from *M. lini*, and a homolog was found for each in *Pgt* (Table 1, Figure 1). Two insertions/deletions were found in PtHSP02-4 and PGTG_3708 (Figure 1A). PtHSP02-5 and PGTG_3709 aligned to homologs from *M. lini*, *Mlp, M. medusae deltoidis*, and *U. maydis*. The N-terminal half of the protein was conserved between *Puccinia* and *Melampsora* (Figure 1B). There appeared to be 48 genus-specific amino acid changes across the protein. *Um* was the most diverged with only a few conserved motifs.

**Figure 2:**
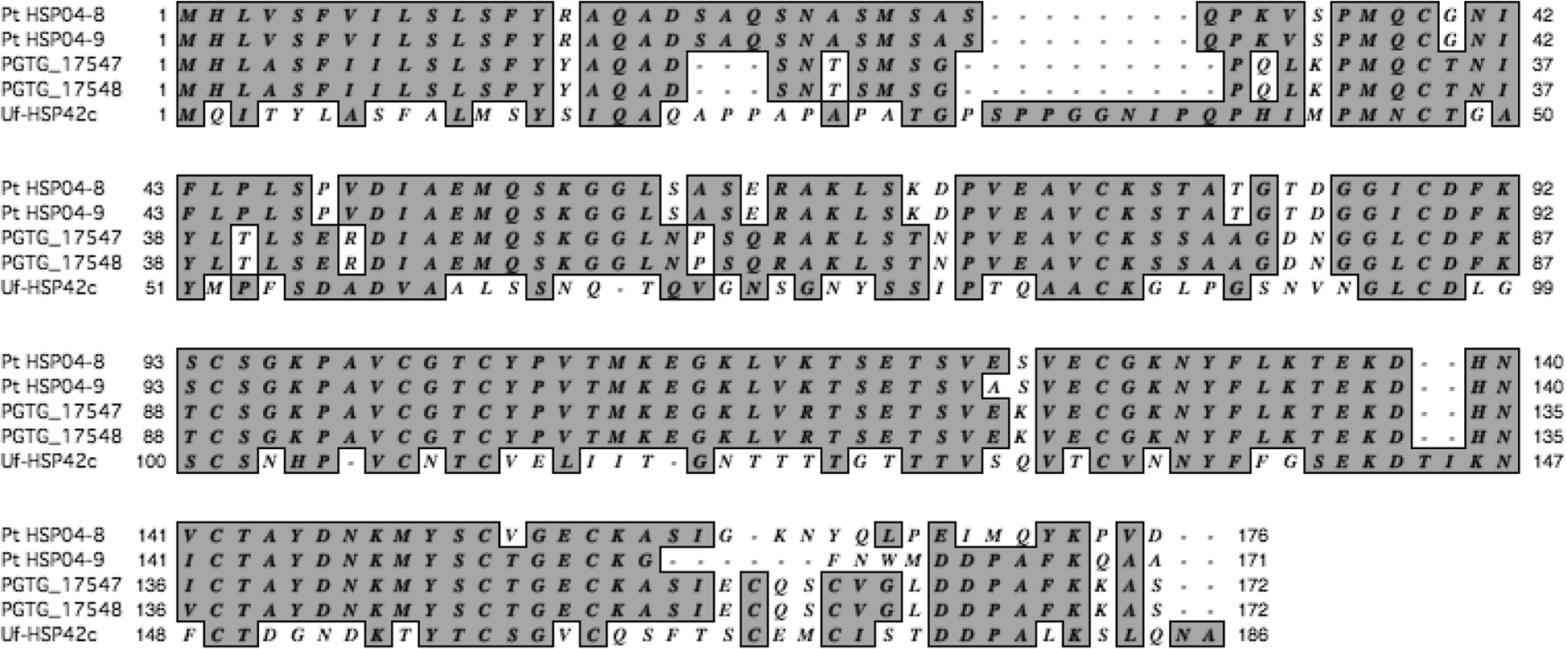
**ClustalW alignments of two predicted secreted proteins coded on BAC clone PtHSP04.** PtHSP04-8 and 9 are aligned to homologs from *Pgt* and *Uromyces fabae*.

Fourteen predicted proteins were identified in PtHSP04 and could be supported through EST sequence alignment. Every protein had a homolog in *Pgt* with protein identities ranging from 26-95% (Table 1); nine could be assigned a putative function. Eight PtHSP04 proteins had homologs in *Mlp* and five in *Um*. PtHSP04-1, 5, and 14 appeared to be unique to *Pt* with little homology to *Pgt*. The predicted transcripts of PtHSP04-6, 7, 8 and 9 aligned to a single EST of *P. striiformis* predicted to encode a secreted protein (ADA54575; [34]) at scores of 4 e^-5^, 2 e^-8^, 6 e^-48^, and 3 e^-9^, respectively. PtHSP04-6 and 7 aligned both to PGTG_17549, though revealing 26 and 60% identity, respectively. The predicted HSP04-7 ORF is 1,095 bp in length and contains a 3’ in-frame repeat of nine nucleotides, GG(C/T) AC(T/A) AC(T/A), translating to 30, three amino acid repeats of Gly-Thr-Thr. Without the repeat, PtHSP04-7 is a homolog to PGTG_17549, while PtHSP04-6 is unique to *Pt*. PtHSP04-8 and 9 are responsible for the homology to Uf-HSP42c and isolation of the BAC clone (Figure 2, Table 1). They are very highly identical except for the C-terminal 18 amino acids, where PtHSP04-9 has a five amino acid deletion and only four identities (Figure 2). Each aligned to PGTG_17547 and PGTG_17548, adjacent proteins which themselves are 100% identical. PtHSP04-8 and 9 are 76% and 71% identical to PGTG_17547, respectively (Figure 2, Table 1).

### Repetitive elements and repeated sequences

Each BAC was evaluated for repeat elements by using REPBASE against *Pgt*, *Pt* and *Pst* genomes. Complete and incomplete terminal inverted repeats (TIR), LTRs, *Copia, Gypsy, Mariner, Mutator, Harbinger*, *Helitron*, hAT, and DNA transposons were found. (Additional file 1: Tables S1 and S2). Major insertions are represented in Figure 1. *Copia* elements were found inserted within *Gypsy* elements in Pt1F16 and PtHSP02. PtHSP02 and PtHSP04 also had localization of LTRs.

### Synteny

**Figure 3:**
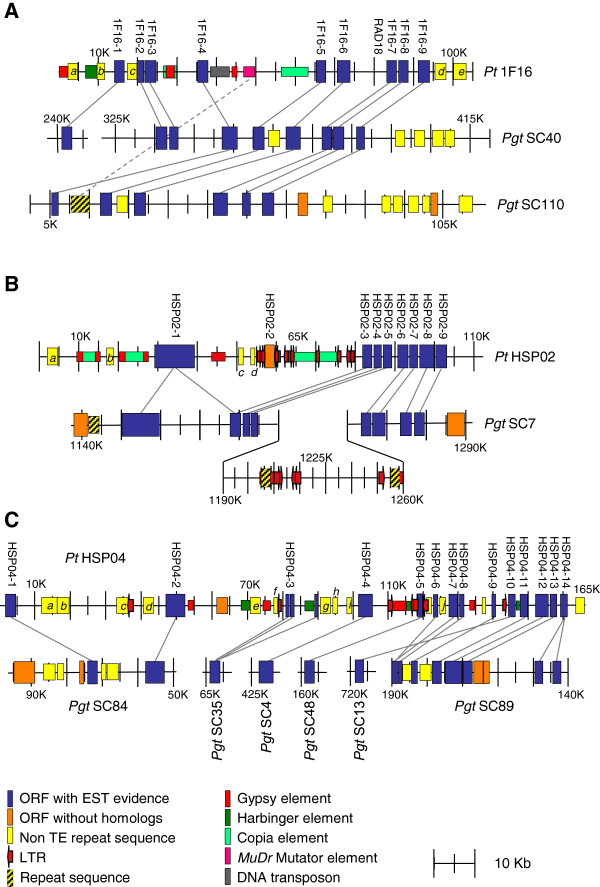
**Graphical representation of three BAC clones from *P. triticina *Pt1F16 (A), PtHSP02 (B) and PtHSP04 (C) and their synteny to super contigs (SC) of *P. graminis tritici *(*Pgt*).** Lines connect homologs between genomes.

To investigate whether the high number of candidate orthologs with *Pgt* maintained the same gene order, the *Pt* BAC sequences were aligned to the available *Pgt* contig sequences. Figure 3 graphically represents the location along each BAC clone of *Pt* ORFs with EST sequence or protein homology support. The majority of Pt1F16 aligned to the 325,000 bp to 415,000 bp region of *Pgt* scaffold (SC) 40 but also to the 5,000 to 65,000 bp region of PgtSC110. PgtSC40 and PgtSC110 could either represent the two *Pgt* haplotypes or a duplication of this region in the genome. Overall, gene order was maintained in both scaffolds. As previously noted, eight of the Pt1F16 ORFs aligned to homologs in *Pgt* but Pt1F16-1 to 3 were found only on PgtSC40 (Table 1, Figure 3A)*.* Pt1F16-1 aligned to PGTG_12990 85 kb upstream in SC40 of PGTG_13012 whereas Pt1F16-2 and 3 were similarly spaced as their counterparts on this *Pgt* SC. Between Pt1F16-4 and 5, four retrotransposons were found, of which one was similar to a retroelement in PgtSC110. No mobile elements were found in this region on PgtSC40. PtRAD18 (Pt1F16-7) is a single ORF while Pt1F16-8 aligned to an ORF corresponding a cysteine rich SCP family protein in both SCs of *Pgt*.

PtHSP02 aligned to a single scaffold, PgtSC7 (Figure 3B). A second haplotype was not detected as the *Pgt* assembly represents most loci with a single sequence [6]. Nine *Pt* ORFs could be aligned to homologs on PgtSC7 (position 1,135,000 to 1,280,000). As with the other BAC clones, gene order was generally maintained. However, PtHSP02-1 and PtHSP02-2 were found embedded between retroelements and LTRs. While PtHSP02-1 aligned to two fragments on PgtSC7, PtHSP02-2 was 48% homologous to a gene on PgtSC15 elsewhere in the genome. The remaining genes in PtHSP02 were in the same order as on PgtSC7, except a large insertion of approximately 70 kB of DNA, including sequence similar to mobile elements, was found between PGTG_03709 and PGTG_03708 on PgtSC7. Additional PgtSC7 DNA insertions were evident within this gene cluster whereas the *Pt* homologs were packed in a tighter arrangement. Across this region, a higher number of retrotransposon elements were found on PtHSP02 (Additional file 1: Table S1).

PtHSP04 aligned to at least six regions within the *Pgt* genome and represents the least syntenic sequence amongst the three BAC clones (Figure 3C). PtHSP04-1 and 2 were found on PgtSC84, however, there were several repeat elements within both the *Pt* and *Pgt* regions. PtHSP04-3 appeared to be a fragmented ORF because a single homologous ORF was found on PgtSC35. PtHSP04-4 and 5 were found on two separate scaffolds, PgtSC4 and PgtSC48, respectively. PtHSP-6, 7, 8, and 9 have homologs on Pgt SC89 in the same order and similar gene distance (conserved micro-synteny). PtHSP04-10, flanked by an LTR and *Harbinger* element, does not have a homolog on PgtSC89, but on PgtSC13. Microsynteny of PtHSP04-11, 12, and 13 to *Pgt* is maintained. PtHSP04-14 is a single copy gene in *Pt* but is repeated in *Pgt*. between BAC positions 60,000 and 125,000 there are a high number repeat elements.

One of the most interesting sets of sequences were *Pt* ORFs for which numerous homologous copies were found in the *Pgt* genome but were not classified as typical mobile elements (identified by small letters in Figure 3; Table 2). Twenty of these ORFs had repeats in the *Pgt* genome numbering from 19 to 474. Table 2 lists the conserved amino acid domains, if present, in each of the ORFs and the percent identity, which. ranged from 34-74%. Each ORF was compared to an RNAseq cDNA library of *Pt*-infected leaf tissue (Fellers and Bruce, unpublished) and nine aligned to the experimental cDNA sequences. The predicted proteins were analyzed for peptide content and most had an abundance of Lys, which is suggestive of helical structures. Each of the proteins was also compared to the PHYRE 2.0 structural data base [35] resulting in seven that revealed regions that aligned, with confidence, to known structures. The first 191 peptides of PtHSP04-j had a structure similar to RAD54, with 99.7% confidence. Of note, PtHSP04-e was expressed and was 51% identical to a protein in *Mlp*.

**Table 2:** **Detailed analysis of non-transposable element, repeated sequences in three *P. triticina *BAC clones**

**ORF**	**Size bp**	***Pgt*repeats**	**Conserved domain**	**% Ident**	Exp*	**Dominant peptide**	**Notes**
1F16-a	757	35	194-443	51	no	9.2% Lys	216-252-40.3% CI Winged helix DNA/RNA binding
1F16-b	458	98	97-458	40	no	11.8% Lys	Highly helical
1F16-c	462	98	all	40	no	13.2% Lys	6-91-60.3% CI ubiquitin ligase
1F-16-d	489	44	344-485	48	no	10.8% Lys	3 alpha helices and 7 beta sheets in conserved domain
						13.0% Ser	
1F16-f	651	80	51-245	74	yes	9.2% Lys	38-163-38.7% CI oxidoreductase
HSP02-a	658	19	all	31	no	9.7% Lys	
HSP02-b	299	80	35-94	68	no	9.4% Lys	32-73-80% CI metal binding protein
						11.7% Ser	145-256-80% CI protease hydrolase inhibitor
HSP02-c	252	50	33-127	52	yes	11.1% Lys	33-95 53% CI DNA binding domain
						10.7% Glu	
HSP02	243	35	all		yes	11.9% Ala	Alignment in *Pgt* are to DNA, not protein
						13.6% Ser	
						11.5% Thr	
HSP04-a	952	74	256-470	48	no	9.9% Ala	
HSP04-b	484	76	300-454	74	no	none	
HSP04-c	442	69	all	34	yes	9.0% Lys	
HSP04-d	679	81	10-351	68	no	9.3% Lys	11 alpha helices in conserved region
HSP04-e	420	131	55-386	55	yes	9.5% ala	216-388-51% identical in Mlp
HSP04-f	262	474	20-189	44	no	11.5% ser	EST sequence hits
HSP04-g	543	96	all	69	yes	11.4% Lys	Highly helical
						9.0% ser	
HSP04-h	262	66	199-258	65	yes	12.6% Lys	P-loop NTPase/DEAD box
HSP04-i	326	20	127-242	34	yes	12.8% Lys	Highly helical
						11.0% Glu	
HSP04-j	309	69	191-303	35	no	9.7% Lys	2-191-99.7% CI Recombinant DNA binding/ RAD54 like
						10.7% Ser	

Each was evaluated for number of repeated homologs in the *Pgt* genome, region of conservation, alignment identity, expression in infected tissue, the dominant peptides, and their best detected fit to a protein structural model.

* Exp - expression in infected tissue 6 dpi.

** PHYRE 2.0 confidence interval (CI) of protein segment matching a structural model with the listed function [35].

## Discussion

This study was performed to look at three regions of the *Pt* genome that were hypothesized to be under selection pressure because of the presence of putative secreted proteins or loci associated with avirulence. To begin with, gene order is conserved between the *Pt* BACs and *Pgt*. However, there is a wide range of protein conservation. A previous comparison of ESTs of Pt and Pgt found a similar level of variation in sequence, but only 40% of the *Pt* EST unigenes had orthologs in *Pgt*[32]. Many genes were likely missing in the unigene set because of the difficulty of sampling other *Pt* life stages to sufficient depth, affecting the percentage. Nevertheless, within the BAC clones, many protein identities were supported by ESTs and similar sequence variation was present [32]. Some proteins were highly conserved between the two wheat rust fungi and had homologs in *Mlp* and *Um*[36].

The three genes used for identifying the BACs were of most interest, in particular, the amount of variation within the sequence. PgtRAD18 had been associated with an avirulence locus in *Pgt*[30]. PtRAD18 protein length is relatively similar but the sequence has diverged from the PgtRAD18 with only 56% identity. Structurally, PtRAD18 is still closely associated with a predicted secreted protein. *Pt* has two genes similar to HESP-379 from *M. lini*[16]. Two indels in PtHSP02-4 suggest a recombination event or splicing difference evolved since the two species diverged, while the sequence differences in the C-terminus of PtHSP02-5 suggest that this region could be very variable. PtHSP04 contained a four-gene locus predicted to code for secreted proteins. Two of them are unique while two are recently duplicated paralogs. Secreted proteins are believed to be most variable amongst fungal proteins because they are under the highest selection pressure to avoid recognition by the host [16,19,37]. At least with these examples, It can be said that sequence variation, recombination, and duplication are driving the changes in these proteins.

Numerous fungal genomes have recently been generated, analyzed, and published. Now comparisons can be made to find core gene families associated with specific life styles and cycles. In an extensive comparison, Duplessis et al. [6] identified core conserved genes needed for biotrophic life in both rust species. It appears that PtHSP02-6 may be one of those genes. PtHSP02-6 aligns with a G-protein beta subunit (GPBS) and no peptide differences were found between *Pt* and *Pgt*. Furthermore, there is little difference between *Pt* and *Mlp* suggesting that this protein is under strong purifying selection in rusts. Yet, the genes flanking PtHSP02-6 are relatively conserved indicating strong selection and the importance of this gene. In *Verticillium dahliae*, mutations in GPBS had reduced virulence, increased microsclerotia and conidiation and decreased ethylene production [38]. GPBS is also involved in similar functions in *F. oxysporum*[39]. In *M. grisea*, GPBS mutants could not form appresorium, and hyphae could not penetrate and grow in rice leaves [40]. The authors also showed that by over expressing GPBS in the fungus, appressorium could form on a hydrophillic surfaces suggesting that GPBS is necessary for control of surface recognition, growth and appressorium formation [40]. Surface recognition and appressorium formation are the key to rust fungal establishment. This suggests that PtHSP02-6 is indispensable for the biotrophic lifecycle and could be a regulating link in pathogenicity.

A strong correlation between genome size and repetitive element content has been found for many fungal genomes. Genome expansion is significant between *Pt* and *Pgt*, even though they are both closely related and are both dikaryotic. The assembled genome for *Pgt* is 89 Mb [6] while *Pt* is currently estimated to be 135 Mb (Broad Institute). The sequence analysis of the three BAC clones gives some indication on why the *Pt* genome may be larger than the *Pgt* genome. Pt1F16 had the least mobile element complexity, but had *Gypsy* elements within *Copia* elements, as did PtHSP02. PtHSP02 also harbored numerous TEs and LTRs in the region between PtHSP02-1 and 3. Meanwhile, PtHSP04 contains more non-TE repeat ORFs, its homologous genes are scattered across *Pgt* scaffolds, and its sequence reveals recombination and/or transposition events disrupting syntenic genes. There is also evidence of gene movement by active elements. PtHSP02-2 was directly flanked by LTRs and was not found in PgtSC7, PtHSP04-5 was also flanked by LTRs and could be found in PgtSC48, and PtHSP04-10 only had a single LTR flanking it, but was flanked on the opposite side by a partial *Harbinger* element. It is possible that since these regions are in repetitive sequence there are assembly errors in *Pgt*, however, each *Pgt* homolog are in high confidence scaffolds.

Most surprising are the non-transposable element, repeated sequences found in the *Pt* BACs (Table 2). Each had homologs throughout the *Pgt* genome. Most had conserved domains that were maintained, while flanking sequences were greatly diverged. Many were high in Lys suggesting a helix protein structure. Some are expressed, based on the presence of an aligning EST, and have homologs in *Mlp*, suggesting an importance. The helical nature of these proteins would suggest their involvement as nucleotide binding elements. *Pt* has five different spore types in its lifecycle involving two different hosts requiring a significant level of cell modifications and cell types. Sequences like these have not been described before and could represent undiscovered elements in the disease cycle.

This work has shown significant genome synteny between two closely related wheat rust fungi. Gene sequences confirmed previous findings of the existence of EST sequence variation between *Pt* and *Pgt.* Various levels of homologies are present, but many of the genes are diverging in a manner that is species specific [32]. Both genomes have a significant amount of mobile elements. Some TE copies are conserved between the two species suggesting ancestral insertion. The insertion of TE sequences helps explain genome expansion, and their insertion near secreted protein genes may alter their regulation or cause their duplication and spread or deletion. Most surprising was the presence of small predicted non-TE genes with numerous homologs in *Pgt*. As many of the small repeated sequences are highly helical in predicted structure, one could suggest they are involved in DNA binding and regulation. Further work is needed to determine when they are expressed and at what stage of the life cycle. When analysis of the *Pt* and *Pst* genomes has been concluded, it can be determined if the repeated nature of these predicted genes is maintained within the wheat rust fungi.

## Methods

### *Pt* BAC library

Total genomic DNA for the BAC library construction was isolated from *P. triticina* (*Pt*) Race1, BBBD [41] urediniospores collected from susceptible wheat (*Triticum aestivum* L.) cultivar Thatcher. Spores were increased on plants spray-inoculated with a urediniospore suspension in light mineral oil (Soltrol 170 isoparaffin, Conoco-Phillips Chemical Co, Borger TX). The oil was allowed to evaporate for 30 min, then plants were moved to a dark dew chamber at 20°C and 100% relative humidity for 24 hrs for urediniospore germination and appressorium formation. Plants were grown in a growth chamber under 16-hour day at 20°C. After 10 days, urediniospores were collected and germinated by densely dusting them over sterile water in dishes for 8 hrs using a volatile nonanol solution (1.56 μl nonanol (Sigma-Aldrich, St. Louis MO), 1 ml acetone, 19 ml of ddH_2_O) spotted on filter paper which was suspended in the lids to stimulate urediniospore germination under crowded conditions. The BAC library was constructed by BioS&T (Montreal, Quebec, Canada; http://www.biost.com). In brief, nuclei were isolated from collected germinated urediniospores and embedded in 1% low melting point agarose plugs. Total genomic DNA embedded in the plugs was partially digested with *Hind*III, separated by electrophoresis by pulse field gel electrophoresis, and the 100–200 kb region was isolated. After electro-elution and dialysis, the DNA fragments were cloned into the *Hind*III site of BAC vector pIndogoBAC5 (Epicenter Technologies, Madison, WI) and propagated in *E. coli* DH10B (Life Technologies, Grand Island, NY).

### BAC clone selection and sequencing

The resulting BAC library of 15,360 individual clones was arrayed on nylon membranes. After colony lysis, DNA was bound to the membranes using standard procedures [41]. BAC filters were probed to identify clones for sequencing. Several candidate fragments were selected as probes. The *Sfi*1 insert from a *Pt* cDNA clone, PT0313.J16.C21 (GenBank accession GR497566; [32]) was labeled with αP^32^-dCTP using a random primer labeling kit (GE Heathcare, Pittsburg, PA). Positive BAC clones were verified by PCR using primers Forward 5’-AGCTCTTCACACGATTCC and Reverse 5’-ATCTTGGCATTGAGCATC. The second probe, SP02, was amplified from *Pt* cDNA clone PT0061b.D10.TB (GenBank accession EC400508) by PCR using primers Forward 5’- CTTTCTAGACCTAGGCAACTTAACAC and Reverse 5’- GCGCCATGGACTAGTTGAAGAGGGA. The third probe, *SP04* was amplified from cDNA clone, PT0131d.B10.BR (GenBank accession EC414978) using PCR primers Forward 5’-CACGAGGGGAACCGATGGGGGT and Reverse 5’-TGGGTTGGTAAACTATTAATGTGCAC. Southern hybridizations were as described [41]

Selected BAC clones were sent as a stab culture to the Genome Center at Washington University, St. Louis, MO. BAC clones were cultured, subcloned, shot gun sequenced, and assembled (Washington University Genome Center, St. Louis, MO). Gene calls were made using FGENESH with gene models specific to *Puccinia* (http://linux1.softberry.com/berry.phtml;). BAC clone gene predictions were compared to *Pgt*, *Mlp* and *Um* genomic resources (http://www.broadinstitute.org/annotation/genome/puccinia_group/Blast.html; http://genomeportal.jgi-psf.org/Mellp1/Mellp1.home.html verified May 7, 2012 and http://www.broadinstitute.org/annotation/genome/ustilago_maydis) using the BLASTN and BLASTX algorithms with settings of E value = 1e^-3^, Matrix = BLOSUM62, and gapped alignment. Repeats were identified using fungaldb of RepBase 17.04 [42], containing the repeats of *Pt, Pgt* and *Pst*. Long terminal repeats (LTR) were determined by LTR_Finder [43] (e.g., red arrows in Figure 3). 

"Mention of a trademark of a proprietary product does not constitute a guarantee of warranty of the product by the United States Department of Agriculture, and does not imply its approval to the exclusion of other products that may also be suitable. USDA is an equal opportunity provider and employer."

## Abbreviations

BAC: Bacterial artificial chromosome; *Pt*: *Puccinia triticina*; *Pgt*: *P. graminis tritici*; *Pst*: *P. stiiformis f.sp tritici*; *Mlp*: *Melampsora larici-populina*; bp: Base pair; HESP: Haustoria expressed secreted protein.

## Competing interests

The authors declare that they have no competing interests.

## Authors’ contributions

GB and JPF conceived the study. GB provided the DNA and constructed the BAC library. BMS and RL designed the primers and isolated the BACs. JPF sequenced the BACs, analyzed the sequence and made gene calls along with MB. CAC analyzed the sequence for repetitive sequence. LS provided the probes for RAD18. JPF, GB, and CAC wrote the manuscript. All authors read and approved the final manuscript.

## Supplementary Material

Additional file 1: Table S1 Repeat elements found in three *P. triticina* BAC clones. BAC sequences were compared to RePBASE, a database containing characterized repeat elements from *P. triticina* (*Pt)*, *P. graminis tritici* (*Pgt*), and *P. striiformis tritici* (*Pst*). Repeats are listed by position in the respective *Pt* BAC clone, DNA strand, and the species specific element. **Table S2.** Description of matching repeats, type of element and which rust fungus they are from. Click here for file

## References

[B1] Cummins GB Hiratsuka Y Illustrated genera of rust fungi 2003 3 St Paul: American Phytophatological Society 225

[B2] Liu JQ Kolmer JA Molecular and virulence diversity and linkage disequilibria in asexual and sexual populations of the wheat leaf rust fungus, puccinia recondita Genome 1998 41 832 840

[B3] Uppalapati S Ishiga Y Doraiswamy V Bedair M Mittal S Chen J Nakashima J Tang Y Tadege M Ratet P Chen R Schultheiss H Mysore KS Loss of abaxial leaf epicutaular wax in *Medicago truncatula irg1/palm1* mutants results in reduced spore differentiation of anthracnose and nonhoast rust pathogens Plant Cell 2012 24 1 353 370 10.1105/tpc.111.093104 22294617 PMC3289574

[B4] Harder JH Bushnell WR, Roelfs AP Developmental ultrastructure of hyphae and spores The cereal rusts, Vol I 1984 Orlando, Florida: Academic 333 373

[B5] Bolton MD Kolmer JA Garvin DF Wheat leaf rust caused by *Puccinia triticina* Mol Plant Path 2008 9 5 563 575 10.1111/j.1364-3703.2008.00487.x 19018988 PMC6640346

[B6] Duplessis S Cuomo C Lin Y-C Aerts A Tisserant E Veneault-Fourrey C Joly DL Hacquard S Amselem J Cantarel BL Chiu R Coutinho PM Feaue N Field M Frey P Gelhaye E Goldberg J Grabherr MG Kodira CD Kohler A Kües U Lindquist EA Lucas SM Mago R Mauceli E Morin E Murat C Pangilinan JL Park R Pearson M Quesneville H Rouhier N Sakthikumar S Salamov AA Schmutz J Selles B Shapiro H Tanguay P Tuskan GA Henrissat B Van de Peer Y Rouzéc P Ellis JG Dodds PN Scheinh JE Zhong S Hameline RC Grigoriev IV Szabo LS Martin F Obligate biotrophy features unraveled by the genomic analysis of rust fungi Proc Nat Acad Sci 2011 108 9166 9171 10.1073/pnas.1019315108 21536894 PMC3107277

[B7] Kasuga T Kozanitas M Bui M Hüberli D Rizzo DM Garbelotto M Phenotypic diversification is associated with host-induced transposon derepression in the sudden oak death pathogen *Phytophthora ramorum* PLoS One 2012 7 4 e34728 10.1371/journal.pone.0034728 22529930 PMC3329494

[B8] Kang S Lebrun MH Farrall L Valent B Gain of virulence caused by insertion of a Pot3 transposon in a *Magnaporthe grisea* avirulence gene MPMI 2001 14 671 674 10.1094/MPMI.2001.14.5.671 11332731

[B9] Gout L Fudal I Kuhn M Blaise F Eckert M Cattolico L Balesdent M-H Rouxel T Lost in the middle of nowhere: the *AvrLm1* avirulence gene of the Dothideomycete *Leptoshaeria maculans* Molec Microb 2006 60 1 67 80 10.1111/j.1365-2958.2006.05076.x 16556221

[B10] Ma L van der Does C Borkovich KA Coleman JJ Daboussi M Comparitive genomics reveals mobile pathogenicity chromosomes in *Fusarium* Nature 2010 64 367 373 20237561 10.1038/nature08850 PMC3048781

[B11] Rep M Kistler HC The genomic organization of plant pathogenicity in *Fusarium* species Curr Opinion in Plant Biol 2011 13 4 420 426 10.1016/j.pbi.2010.04.004 20471307

[B12] Kämper J Kahmann R Bölker M Ma LJ Brefort T Saville BJ Banuett F Kronstad JW Gold SE Müller O Perlin MH Wösten HA de Vries R Ruiz-Herrera J Reynaga-Peña CG Snetselaar K McCann M Pérez-Martín J Feldbrügge M Basse CW Steinberg G Ibeas JI Holloman W Guzman P Farman M Stajich JE Sentandreu R González-Prieto JM Kennell JC Molina L Schirawski J Mendoza-Mendoza A Greilinger D Münch K Rössel N Scherer M Vranes M Ladendorf O Vincon V Fuchs U Sandrock B Meng S Ho EC Cahill MJ Boyce KJ Klose J Klosterman SJ Deelstra HJ Ortiz-Castellanos L Li W Sanchez-Alonso P Schreier PH Häuser-Hahn I Vaupel M Koopmann E Friedrich G Voss H Schlüter T Margolis J Platt D Swimmer C Gnirke A Chen F Vysotskaia V Mannhaupt G Güldener U Münsterkötter M Haase D Oesterheld M Mewes HW Mauceli EW DeCaprio D Wade CM Butler J Young S Jaffe DB Calvo S Nusbaum C Galagan J Birren BW Insights from the genome of the biotrophic fungal plant pathogen *Ustilago maydis* Nature 2006 444 7115 97 101 10.1038/nature05248 17080091

[B13] Cuomo C Güldener U Xu JR Trail F Turgeon BG Di Pietro A Walton JD Ma L Baker SE Rep M Adam G Antoniw J Baldwin T Calvo S Chang Y DeCaprio D Gale LR Gnerre S Goswami RS Hammond-Kosack K Harris LJ Hilburn K Kennell JC Kroken S Magnuson JK Mannhaupt G Mauceli E Mewes H Mitterbauer R Muehlbauer G Münsterkötter M Nelson D O'Donnell K Ouellet T Qi W Quesneville H Roncero MIG Seong K Tetko IV Urban M Waalwijk C Ward TJ Ao J Birren BW Kistler HC The *Fusarium graminearum* genome reveals a link between localized polymorphism and pathogen specialization Science 2007 317 5843 1400 1402 10.1126/science.1143708 17823352

[B14] Dean RA Talbot NJ Ebbole DJ Farman ML Mitchell TK Orbach MJ Thon M Kulkarni R Xu JR Pan H Read ND Lee YH Carbone I Brown D Oh YY Donofrio N Jeong JS Soanes DM Djonovic S Kolomiets E Rehmeyer C Li W Harding M Kim S Lebrun MH Bohnert H Coughlan S Butler J Calvo S Ma LJ Nicol R Purcell S Nusbaum C Galagan JE Birren BW The genome sequence of the rice blast fungus *Magnaporthe grisea* Nature 2005 434 7036 980 986 10.1038/nature03449 15846337

[B15] Dodds PN Lawrence GJ Catanzariti AM Teh T Wang CIA Ayliffe MA Kobe B Ellis JG Direct protein interaction underlies gene-for-gene specificity and coevolution of the flax resistance genes and flax rust avirulence genes Proc Nat Acad Sci USA 2006 103 8888 8893 10.1073/pnas.0602577103 16731621 PMC1482673

[B16] Catanzariti AM Dodds PN Lawrence GJ Ayliffe MA Ellis JG Haustorially expressed secreted proteins from flax rust are highly enriched for avirulence elicitors Plant Cell 2006 18 243 256 10.1105/tpc.105.035980 16326930 PMC1323496

[B17] Nirmala J Drader T Lawrence PK Yin C Hulbert S Steber CM Steffenson BJ Szabo LJ von Wettstein D Kleinhofs A Conerted action of two avirulent spore effectors activates *Reaction to Puccinia graminis 1* (*Rpg1*)-mediated cereal stem rust resistance Proc Nat Acad Sci USA 2011 108 35 14676 14681 10.1073/pnas.1111771108 21873196 PMC3167542

[B18] Spanu PD Abbott JC Amselem J Burgis TA Soanes DM Stüber K Ver Loren Van Themaat E Brown JK Butcher SA Gurr SJ Lebrun MH Ridout CJ Schulze-Lefert P Talbot NJ Ahmadinejad N Ametz C Barton GR Benjdia M Bidzinski P Bindschedler LV Both M Brewer MT Cadle-Davidson L Cadle-Davidson MM Collemare J Cramer R Frenkel O Godfrey D Harriman J Hoede C King BC Klages S Kleemann J Knoll D Koti PS Kreplak J López-Ruiz FJ Lu X Maekawa T Mahanil S Micali C Milgroom MG Montana G Noir S O'Connell RJ Oberhaensli S Parlange F Pedersen C Quesneville H Reinhardt R Rott M Sacristán S Schmidt SM Schön M Skamnioti P Sommer H Stephens A Takahara H Thordal-Christensen H Vigouroux M Wessling R Wicker T Panstruga R Genome expansion and gene loss in powdery mildew fungi reveal tradeoffs in extreme parasitism Science 2010 330 6010 1543 15436 10.1126/science.1194573 21148392

[B19] Schirawski J Mannhaupt G Münch K Brefort T Schipper K Doehlemann G Stasio MD Rössel N Mendoza-Mendoza A Pester D Müller O Winterberg B Meyer E Ghareeb H Wollenbert T Münsterkötter M Wong P Walter M Stukenbrock E Güldener U Kahmann R Pathogenicity determinants in smut fungi revealed by genome comparison Science 2010 330 1546 1548 10.1126/science.1195330 21148393

[B20] Jin Y Szabo LJ Carson M Century-old mystery of *Puccinia striiformis* life history solved with the identification of *Berberis* as an alternate host Phytopathology 2010 100 432 435 10.1094/PHYTO-100-5-0432 20373963

[B21] Saari EE Young HC Kernkamp MF Infection of North American *Thalictrum ssp*. with *Puccinia recondita f.sp. tritici* Phytopathology 1968 58 939 943

[B22] Chester KS The Nature And Prevention Of The Cereal Rusts As Exemplified In The Leaf Rust Of Wheat 1946 Waltham Mass: Chronica Botanica Co

[B23] Jackson HS Mains EB Aecial stage of the orange leaf rust of wheat, *Puccinia triticina* Eriks Journal of Agr Res 1921 22 151 172

[B24] Goyeau H Halkett F Zapater M Carlier J Lannou C Clonality and host selection in the wheat pathogenic fungus *Puccinia triticina* Fungal Genetics and Biol 2007 44 6 474 483 10.1016/j.fgb.2007.02.006 17412619

[B25] Kolmer JA Liu JQ Sies M Virulence and molecular polymorphism in *Puccinia recondita* fsp *tritici* in Canada Phytopathology 1995 85 276 285 10.1094/Phyto-85-276

[B26] Kolmer JA Ordoñez ME Genetic differentiation of *Puccinia triticina* populations in central Asia and the Caucasus Phytopathology 2007 97 1141 1149 10.1094/PHYTO-97-9-1141 18944179

[B27] Ordoñez ME Kolmer JA Differentiation of molecular genotypes and virulence phenotypes of *Puccinia triticina* from common wheat in North America Phytopathology 2009 99 750 758 10.1094/PHYTO-99-6-0750 19453235

[B28] Ordoñez ME German SE Kolmer JA Genetic differentiation within the *Puccinia triticina* population in South America and comparison with the North American population suggests common ancestry and intercontinental migration Phytopathology 2010 100 376 383 10.1094/PHYTO-100-4-0376 20205541

[B29] Wang X McCallum B Fusion body formation, germ tube anastomosis, and nuclear migration during the germination of urediniospores of the wheat leaf rust fungus, *Puccinia triticina* Phytopathology 2009 99 12 1355 1364 10.1094/PHYTO-99-12-1355 19900001

[B30] Zambino PJ Kubelik AR Szabo LJ Gene action and linkage of avirulence genes to DNA markers in the rust fungus *Puccinia graminis* Phytopathology 2000 90 819 826 10.1094/PHYTO.2000.90.8.819 18944502

[B31] Hu G Linning R McCallum B Banks T Cloutier S Butterfield Y Liu J Kirkpatrick R Stott J Yang G Smailus D Jones S Marra M Schein J Bakkeren G Generation of a wheat leaf rust, *Puccinia triticina*, EST database from stage-specific cDNA libraries Mol Plant Pathol 2007 8 451 467 10.1111/j.1364-3703.2007.00406.x 20507513

[B32] Xu J Linning R Fellers J Dickinson M Zhu W Antonov I Joly D Donaldson ME Eilam T Anikster Y Banks T Munro S Mayo M Wynhoven B Ali J Moore R McCallum B Borodovsky M Saville B Bakkeren G Gene discovery in EST sequences from the wheat leaf rust fungus Puccinia triticina sexual spores, asexual spores and haustoria, compared to other rust and corn smut fungi BMC Genomics 2011 12 161 10.1186/1471-2164-12-161 21435244 PMC3074555

[B33] Link TI Voegele RT Secreted proteins of *Uromyces fabae*: similarities and stage specificity Mol Plant Pathology 2008 9 1 59 66 10.1111/j.1364-3703.2007.00448.x PMC6640452 18705884

[B34] Dong YL Yin CT Hulbert S Chen XM Kang ZS Cloning and expression analysis of three secreted protein genes from wheat stripe rust fungus *Puccinia striiformis f. sp. tritici* J Microbiol Biotechnol 2011 27 5 1261 1265 10.1007/s11274-010-0565-6

[B35] Kelley LA Sternberg MJE Protein structure prediction on the web: a case study using the Phyre server Nat Protoc 2009 4 363 371 19247286 10.1038/nprot.2009.2

[B36] Oberhaensli S Parlange F Buchmann JP Jenny FH Abbott JC Burgis TA Spanu PD Keller B Wicker T Comparative sequence analysis of wheat and barley powdery mildew fungi reveals colinearity, dates of divergence and indicates host-pathogen co-evolution Fungal Gen Biol 2011 48 327 334 10.1016/j.fgb.2010.10.003 20955813

[B37] Laurie JD Ali S Linning R Mannhaupt G Wong P Güldener U Münsterkötter M Link TI Voegele RT Secreted proteins of *Uromyces fabae*: similarities and stage specificity Mol Plant Pathol 2008 9 59 66 18705884 10.1111/j.1364-3703.2007.00448.x PMC6640452

[B38] Tzima AK Paplomatas EJ Tsitsigiannis DI Kang S The G protein ß subunit controls virulence and multiple growth- and development -related traits in *Verticillium dahlia* Fungal Genet Biol 2012 49 4 271 283 10.1016/j.fgb.2012.02.005 22387367

[B39] Delgado-Jarana J Martínez-Rocha AL Roldán-Rodriguez R Roncero MI Di Pietro A *Fusarium oxysporum* G-protein beta subunit Fgb1 regulates hyphal growth, development, and virulence through multiple signalling pathways Fungal Genet Biol 2005 42 1 61 72 10.1016/j.fgb.2004.10.001 15588997

[B40] Nishimura M Park G Xu JR The G-beta subunit MGB1 is involved in regulating multiple steps of infection-related morphogenesis in *Magnaporthe grisea* Mol Microbiol 2003 50 1 231 243 10.1046/j.1365-2958.2003.03676.x 14507377

[B41] Sambrook J Rusell DW Molecular Cloning: A Laboratory Manual 2001 3 Cold Spring Harbor: NY: Cold Spring Harbor Laboratory Press

[B42] Jurka J Kapitonov VV Pavlicek A Klonowski P Kohany O Walichiewicz J Repbase Update, a database of eukaryotic repetitive elements Cytogentic and Genome Res 2005 110 462 467 10.1159/000084979 16093699

[B43] Xu Z Wang H LTR_FINDER: an efficient tool for the prediciton of full-length LTR retrotransposons Nucleic Acids Res 2007 35 W265 W268 10.1093/nar/gkm286 17485477 PMC1933203

