# Supplementary material for: Conserved loci of leaf and stem rust fungi of wheat share synteny interrupted by lineage-specific influx of repeat elements
Source: BMC Genomics. 2013 Jan 29;14:60. doi: 10.1186/1471-2164-14-60 (PMC3579696; doi:10.1186/1471-2164-14-60)
Supplement: Additional file 1: Table S1 — Repeat elements found in three P. triticina BAC clones. BAC sequences were compared to RePBASE, a database containing characterized repeat elements from P. triticina (Pt), P. graminis tritici (Pgt), and P. striiformis tritici (Pst). Repeats are listed by position in the respective Pt BAC clone, DNA strand, and the species specific element. Table S2. Description of matching repeats, type of element and which rust fungus they are from. [file 1471-2164-14-60-S1.docx]

Supplemental Table 1. Repeat elements found in three *P. triticina* BAC clones. BAC sequences were compared to RePBASE, a database containing characterized repeat elements from *P. triticina* (*Pt)*, *P. graminis tritici* (*Pgt*), and *P. striiformis* *tritici* (*Pst*). Repeats are listed by position in the respective *Pt* BAC clone, DNA strand, and the species specific element.

|  | Position in sequence | |  |  |
| --- | --- | --- | --- | --- |
| BAC | Begin | End | Strand | Matching repeat |
| Pt1F16 | 1695 | 1886 | C | Gypsy-2_Pgt-LTR |
| Pt1F16 | 2743 | 2901 | C | Gypsy-7_Pgt-I |
| Pt1F16 | 3013 | 3554 | C | Gypsy-8_Pst-I |
| Pt1F16 | 4473 | 4569 | C | Gypsy-1_Pst-LTR |
| Pt1F16 | 6260 | 6322 | + | (CAAAAA)n |
| Pt1F16 | 6715 | 6993 | C | DIRS-1_Pt |
| Pt1F16 | 7766 | 7821 | C | DNA-29_Pst |
| Pt1F16 | 7821 | 7864 | + | Harbinger-1_Pst |
| Pt1F16 | 8756 | 8912 | + | Harbinger-16_Pgt |
| Pt1F16 | 9580 | 9801 | + | Harbinger-16_Pgt |
| Pt1F16 | 14019 | 14128 | + | DNA-13_Pst |
| Pt1F16 | 14034 | 14141 | + | DNA-13_Pst |
| Pt1F16 | 17171 | 17200 | C | (CAA)n |
| Pt1F16 | 25260 | 25347 | + | DNA8-1_Pgt |
| Pt1F16 | 28203 | 28588 | C | Copia-14_Pgt-I |
| Pt1F16 | 28648 | 28892 | + | Gypsy-12_Pgt-LTR |
| Pt1F16 | 29057 | 29119 | + | Gypsy-12_Pgt-LTR |
| Pt1F16 | 29244 | 29338 | + | Gypsy-12_Pgt-I |
| Pt1F16 | 29422 | 29639 | + | Gypsy-12_Pgt-I |
| Pt1F16 | 29974 | 30321 | C | Copia-14_Pgt-LTR |
| Pt1F16 | 30367 | 30390 | + | DNA7-3_Pt |
| Pt1F16 | 30391 | 30418 | + | (CGTAG)n |
| Pt1F16 | 30419 | 30797 | + | DNA7-3_Pt |
| Pt1F16 | 30634 | 30838 | + | DNA7-3_Pt |
| Pt1F16 | 32120 | 32205 | C | REP-15_Pst |
| Pt1F16 | 38382 | 38468 | + | DNA7-1F_Pgt |
| Pt1F16 | 38720 | 38867 | C | hAT-5_Pst |
| Pt1F16 | 38886 | 39035 | + | DNA8-1_Pgt |
| Pt1F16 | 38913 | 39057 | + | DNA8-1_Pgt |
| Pt1F16 | 39230 | 39516 | C | DNA8-1_Pgt |
| Pt1F16 | 39305 | 39635 | + | DNA8-1_Pgt |
| Pt1F16 | 39355 | 39690 | + | DNA8-1_Pgt |
| Pt1F16 | 39402 | 39736 | C | DNA8-1_Pgt |
| Pt1F16 | 39493 | 39827 | C | DNA8-1_Pgt |
| Pt1F16 | 39539 | 39874 | + | DNA8-1_Pgt |
| Pt1F16 | 39581 | 39920 | + | DNA8-1_Pgt |
|  | Position in sequence | |  |  |
| BAC | Begin | End | Strand | Matching repeat |
| Pt1F16 | 39625 | 39966 | C | DNA8-1_Pgt |
| Pt1F16 | 39667 | 40012 | + | DNA8-1_Pgt |
| Pt1F16 | 39713 | 40058 | C | DNA8-1_Pgt |
| Pt1F16 | 39763 | 40104 | C | DNA8-1_Pgt |
| Pt1F16 | 39850 | 40160 | + | DNA8-1_Pgt |
| Pt1F16 | 39942 | 40288 | C | DNA8-1_Pgt |
| Pt1F16 | 39988 | 40334 | + | DNA8-1_Pgt |
| Pt1F16 | 40034 | 40400 | C | DNA8-1_Pgt |
| Pt1F16 | 40126 | 40447 | + | DNA8-1_Pgt |
| Pt1F16 | 40172 | 40493 | + | DNA8-1_Pgt |
| Pt1F16 | 40218 | 40585 | C | DNA8-1_Pgt |
| Pt1F16 | 40388 | 40723 | C | DNA8-1_Pgt |
| Pt1F16 | 40423 | 40769 | + | DNA8-1_Pgt |
| Pt1F16 | 40469 | 40815 | C | DNA8-1_Pgt |
| Pt1F16 | 40515 | 40861 | C | DNA8-1_Pgt |
| Pt1F16 | 40561 | 40907 | C | DNA8-1_Pgt |
| Pt1F16 | 40607 | 40952 | + | DNA8-1_Pgt |
| Pt1F16 | 40653 | 40999 | C | DNA8-1_Pgt |
| Pt1F16 | 40699 | 41045 | C | DNA8-1_Pgt |
| Pt1F16 | 40745 | 41091 | C | DNA8-1_Pgt |
| Pt1F16 | 40791 | 41137 | + | DNA8-1_Pgt |
| Pt1F16 | 40883 | 41229 | C | DNA8-1_Pgt |
| Pt1F16 | 40929 | 41275 | C | DNA8-1_Pgt |
| Pt1F16 | 41021 | 41367 | C | DNA8-1_Pgt |
| Pt1F16 | 41067 | 41413 | C | DNA8-1_Pgt |
| Pt1F16 | 41113 | 41459 | + | DNA8-1_Pgt |
| Pt1F16 | 41159 | 41504 | C | DNA8-1_Pgt |
| Pt1F16 | 41205 | 41551 | C | DNA8-1_Pgt |
| Pt1F16 | 41251 | 41597 | C | DNA8-1_Pgt |
| Pt1F16 | 41297 | 41643 | C | DNA8-1_Pgt |
| Pt1F16 | 41343 | 41689 | + | DNA8-1_Pgt |
| Pt1F16 | 41435 | 41781 | + | DNA8-1_Pgt |
| Pt1F16 | 41481 | 41827 | C | DNA8-1_Pgt |
| Pt1F16 | 41527 | 41873 | C | DNA8-1_Pgt |
| Pt1F16 | 41573 | 41919 | C | DNA8-1_Pgt |
| Pt1F16 | 41619 | 41965 | C | DNA8-1_Pgt |
| Pt1F16 | 41665 | 42010 | + | DNA8-1_Pgt |
| Pt1F16 | 41711 | 42057 | C | DNA8-1_Pgt |
| Pt1F16 | 41757 | 42103 | C | DNA8-1_Pgt |
| Pt1F16 | 41803 | 42149 | C | DNA8-1_Pgt |
| Pt1F16 | 41849 | 42195 | + | DNA8-1_Pgt |
|  | Position in sequence | |  |  |
| BAC | Begin | End | Strand | Matching repeat |
| Pt1F16 | 41941 | 42287 | C | DNA8-1_Pgt |
| Pt1F16 | 41987 | 42333 | C | DNA8-1_Pgt |
| Pt1F16 | 42033 | 42379 | + | DNA8-1_Pgt |
| Pt1F16 | 42079 | 42424 | C | DNA8-1_Pgt |
| Pt1F16 | 43032 | 43083 | C | (CAAA)n |
| Pt1F16 | 43536 | 43718 | C | DNA3-1_Pst |
| Pt1F16 | 43725 | 43846 | + | Harbinger-N1_Pt |
| Pt1F16 | 43978 | 44010 | C | AT_rich |
| Pt1F16 | 44449 | 44615 | C | Gypsy-12_Pgt-I |
| Pt1F16 | 44544 | 44664 | C | REP-5_Pst |
| Pt1F16 | 44949 | 45049 | C | Gypsy-12_Pgt-LTR |
| Pt1F16 | 45399 | 45626 | C | REP-5_Pst |
| Pt1F16 | 45625 | 45662 | C | Gypsy-12_Pgt-I |
| Pt1F16 | 48500 | 48660 | + | MuDR-3_Pgt |
| Pt1F16 | 48571 | 48674 | + | MuDR-2_Pst |
| Pt1F16 | 50261 | 50335 | + | MuDR-3_MLP |
| Pt1F16 | 50852 | 50872 | C | AT_rich |
| Pt1F16 | 52713 | 52835 | + | DNA8-1_Pgt |
| Pt1F16 | 53020 | 53117 | + | REP-1_Pst |
| Pt1F16 | 53085 | 53142 | C | Harbinger-N1_Pst |
| Pt1F16 | 53222 | 53244 | + | (TA)n |
| Pt1F16 | 53277 | 53413 | + | REP-1_Pst |
| Pt1F16 | 53312 | 53534 | + | REP-1_Pst |
| Pt1F16 | 54250 | 54387 | C | hAT-5_Pst |
| Pt1F16 | 54502 | 54808 | + | DNA8-1_Pgt |
| Pt1F16 | 54702 | 54970 | + | DNA8-1_Pgt |
| Pt1F16 | 55095 | 55309 | C | LTR-1_Pt-LTR |
| Pt1F16 | 55310 | 55516 | C | LTR-1_Pt-I |
| Pt1F16 | 55517 | 55731 | C | LTR-1_Pt-LTR |
| Pt1F16 | 55731 | 55810 | C | DNA8-1_Pgt |
| Pt1F16 | 57333 | 57356 | C | AT_rich |
| Pt1F16 | 57550 | 58001 | + | Copia-17_Pgt-LTR |
| Pt1F16 | 58001 | 58031 | + | Copia-32_MLP-I |
| Pt1F16 | 58305 | 59388 | + | Copia-17_Pgt-I |
| Pt1F16 | 59725 | 62789 | + | Copia-17_Pgt-I |
| Pt1F16 | 62842 | 63317 | + | Copia-17_Pgt-LTR |
| Pt1F16 | 63357 | 63427 | + | Harbinger-N1_Pt |
| Pt1F16 | 63622 | 63703 | C | DNA3-6_Pst |
| Pt1F16 | 63826 | 63936 | + | DNA2-2_Pst |
| Pt1F16 | 68647 | 68708 | C | EnSpm-N1_Pst |
| Pt1F16 | 68865 | 69341 | + | DNA7-3_Pt |
|  | Position in sequence | |  |  |
| BAC | Begin | End | Strand | Matching repeat |
| Pt1F16 | 69560 | 69671 | C | EnSpm-N1_Pst |
| Pt1F16 | 69666 | 69745 | C | DNA-30_Pst |
| Pt1F16 | 70503 | 70568 | C | DNA3-16_Pst |
| Pt1F16 | 70565 | 70705 | + | DNA3-19_Pst |
| Pt1F16 | 70706 | 70793 | + | DNA3-16_Pst |
| Pt1F16 | 75555 | 75636 | C | Gypsy-4_Pt-I |
| Pt1F16 | 76047 | 79180 | C | hAT-5_Pst |
| Pt1F16 | 79706 | 79798 | C | DNA3-2_Pt |
| Pt1F16 | 85614 | 85698 | + | DNA7-2_Pgt |
| Pt1F16 | 86886 | 88154 | + | Tad1-1_Pt |
| Pt1F16 | 88348 | 88466 | C | DNA-13_Pst |
| Pt1F16 | 88363 | 88483 | + | DNA-13_Pst |
| Pt1F16 | 88563 | 88624 | + | DNA3-2_Pt |
| Pt1F16 | 88580 | 88664 | + | DNA3-2_Pt |
| Pt1F16 | 88613 | 88839 | + | DNA3-2_Pt |
| Pt1F16 | 88791 | 88869 | + | DNA3-2_Pt |
| Pt1F16 | 89114 | 89214 | + | DNA-13_Pst |
| Pt1F16 | 89318 | 89467 | + | DNA3-16_Pst |
| Pt1F16 | 90136 | 90235 | + | DNA-13_Pst |
| Pt1F16 | 90166 | 90323 | + | DNA-13_Pst |
| Pt1F16 | 97254 | 97305 | + | DNA-13_Pst |
| Pt1F16 | 97285 | 97382 | C | DNA7-1F_Pgt |
| Pt1F16 | 97314 | 97518 | C | DNA7-3_Pt |
| Pt1F16 | 97355 | 97732 | C | DNA7-3_Pt |
| Pt1F16 | 97733 | 97760 | C | (CGTAG)n |
| Pt1F16 | 97761 | 97784 | C | DNA7-3_Pt |
| Pt1F16 | 97788 | 97855 | + | DNA-22_Pst |
| Pt1F16 | 97812 | 97869 | C | DNA-13_Pst |
| Pt1F16 | 98504 | 98651 | + | Harbinger-N1_Pst |
| Pt1F16 | 98748 | 98868 | + | DNA-13_Pst |
| Pt1F16 | 98792 | 98938 | + | DNA-13_Pst |
| Pt1F16 | 98973 | 99151 | + | DNA3-2_Pt |
| Pt1F16 | 99276 | 99388 | + | Harbinger-10_Pst |
| Pt1F16 | 101398 | 101418 | C | AT_rich |
| Pt1F16 | 101992 | 102579 | C | REP-16_Pst |

Supplemental Table 1 continued.

|  | Position in sequence | |  |  |
| --- | --- | --- | --- | --- |
| BAC | Begin | End | Strand | Matching repeat |
| PtHSP2 | 6555 | 6578 | C | AT_rich |
| PtHSP2 | 7333 | 7419 | C | (GAAA)n |
| PtHSP2 | 8321 | 9613 | C | Mariner-4_Pt |
| PtHSP2 | 9808 | 9857 | C | (CAAAA)n |
| PtHSP2 | 10256 | 10304 | + | LTR-4_Pst-LTR |
| PtHSP2 | 10689 | 10769 | + | Gypsy-12_Pgt-LTR |
| PtHSP2 | 10913 | 10986 | + | REP-5_Pst |
| PtHSP2 | 10924 | 11043 | + | Gypsy-12_Pgt-I |
| PtHSP2 | 12369 | 12612 | + | Copia-9_Pgt-LTR |
| PtHSP2 | 12850 | 13428 | + | Copia-9_Pgt-I |
| PtHSP2 | 13855 | 15152 | + | Copia-9_Pgt-I |
| PtHSP2 | 15274 | 15341 | + | Copia-9_Pgt-I |
| PtHSP2 | 15395 | 15525 | C | (GGGAGA)n |
| PtHSP2 | 15566 | 15844 | + | Gypsy-12_Pgt-LTR |
| PtHSP2 | 15922 | 16008 | + | Gypsy-12_Pgt-LTR |
| PtHSP2 | 16314 | 16466 | + | Gypsy-12_Pgt-I |
| PtHSP2 | 16558 | 16895 | + | Copia-9_Pgt-I |
| PtHSP2 | 20976 | 21011 | + | Gypsy-12_Pgt-I |
| PtHSP2 | 21114 | 21209 | C | Gypsy-2_Pgt-I |
| PtHSP2 | 22149 | 22481 | + | hAT-4_Pst |
| PtHSP2 | 23527 | 23793 | C | Copia-48_MLP-I |
| PtHSP2 | 23738 | 23876 | C | Copia-10_Pgt-I |
| PtHSP2 | 23937 | 24103 | C | Copia-12_Pgt-I |
| PtHSP2 | 24234 | 24674 | C | Copia-5_Pgt-I |
| PtHSP2 | 24594 | 24704 | C | Copia-11_Pgt-I |
| PtHSP2 | 25515 | 25867 | C | Copia-11_Pgt-I |
| PtHSP2 | 25570 | 25915 | C | Copia-16_Pgt-I |
| PtHSP2 | 27483 | 27651 | + | DNA7-1D_Pgt |
| PtHSP2 | 27508 | 27833 | + | DNA7-2_Pt |
| PtHSP2 | 28415 | 28463 | + | hAT-N1_Pgt |
| PtHSP2 | 28454 | 28513 | + | Gypsy-12_Pgt-I |
| PtHSP2 | 29198 | 29306 | + | (GAAA)n |
| PtHSP2 | 37273 | 37480 | + | LTR-1_Pt-LTR |
| PtHSP2 | 43257 | 43326 | + | Gypsy-7_Pgt-LTR |
| PtHSP2 | 44077 | 44231 | C | Gypsy-12_Pgt-I |
| PtHSP2 | 44165 | 44419 | C | REP-5_Pst |
| PtHSP2 | 44808 | 45014 | C | REP-5_Pst |
| PtHSP2 | 45037 | 45783 | + | Gypsy-1_Pst-I |
|  | Position in sequence | |  |  |
| BAC | Begin | End | Strand | BAC |
| PtHSP2 | 45818 | 46962 | + | Gypsy-1_Pst-I |
| PtHSP2 | 47283 | 47535 | C | DNA-27_Pst |
| PtHSP2 | 48582 | 48796 | C | DNA3-9_Pst |
| PtHSP2 | 49318 | 49596 | + | Gypsy-4_Pt-I |
| PtHSP2 | 51329 | 51600 | + | DNA7-2_Pt |
| PtHSP2 | 51601 | 51626 | + | (CGTAG)n |
| PtHSP2 | 51627 | 51794 | + | DNA7-2_Pt |
| PtHSP2 | 52082 | 52504 | C | DNA7-2_Pt |
| PtHSP2 | 53789 | 54803 | + | Gypsy-4_Pt-I |
| PtHSP2 | 54917 | 55157 | + | Gypsy-4_Pt-I |
| PtHSP2 | 55215 | 55413 | + | Harbinger-N1_Pt |
| PtHSP2 | 55422 | 55451 | C | (A)n |
| PtHSP2 | 57717 | 57748 | C | Helitron-1_Pgt |
| PtHSP2 | 58208 | 58321 | + | P-N1_Pt |
| PtHSP2 | 61908 | 62840 | C | Gypsy-15_Pgt-LTR |
| PtHSP2 | 65287 | 65349 | + | Copia-10_Pgt-I |
| PtHSP2 | 65747 | 65973 | + | Copia-1_Pgt-I |
| PtHSP2 | 66329 | 66396 | + | Copia-5_Pgt-LTR |
| PtHSP2 | 66719 | 67571 | + | Copia-6_Pgt-I |
| PtHSP2 | 66827 | 68694 | + | Copia-2_Pgt-I |
| PtHSP2 | 69217 | 69323 | + | (GAAAA)n |
| PtHSP2 | 69442 | 69776 | + | Copia-1_Pgt-I |
| PtHSP2 | 69777 | 70101 | + | REP-2_Pst |
| PtHSP2 | 70328 | 70509 | C | Harbinger-1_Pst |
| PtHSP2 | 70451 | 71660 | C | Copia-5_Pst-I |
| PtHSP2 | 70620 | 73223 | C | Copia-3_Pst-I |
| PtHSP2 | 73507 | 73876 | C | Copia-2_Pst-I |
| PtHSP2 | 75510 | 75614 | + | (GGGAA)n |
| PtHSP2 | 76181 | 76317 | C | Harbinger-1_Pst |
| PtHSP2 | 76318 | 76409 | C | (TAG)n |
| PtHSP2 | 76432 | 76588 | C | REP-13_Pst |
| PtHSP2 | 77358 | 77763 | + | Copia-11_Pt-I |
| PtHSP2 | 77782 | 77824 | + | Copia-5_Pgt-LTR |
| PtHSP2 | 80541 | 80603 | C | DNA3-8_Pst |
| PtHSP2 | 80602 | 80645 | + | Gypsy-4_Pt-I |
| PtHSP2 | 81264 | 81354 | C | DNA-30_Pst |
| PtHSP2 | 83611 | 83741 | + | DNA3-16_Pst |
| PtHSP2 | 89742 | 89813 | + | P-N1_Pt |
| PtHSP2 | 92412 | 92453 | + | (TGGGGG)n |
| PtHSP2 | 93081 | 93119 | + | (A)n |
| PtHSP2 | 101223 | 101247 | C | (CAA)n |
|  | Position in sequence | |  |  |
| BAC | Begin | End | Strand | BAC |
| PtHSP2 | 102560 | 102657 | C | Gypsy-1_Pt-LTR |
| PtHSP2 | 106613 | 106706 | C | Harbinger-10_Pgt |
| PtHSP2 | 106632 | 106735 | C | Harbinger-18_Pgt |
| PtHSP2 | 107574 | 107767 | C | Mariner-2_Pst |
| PtHSP2 | 108379 | 108565 | + | DNA8-1_Pgt |
| PtHSP2 | 108940 | 109134 | + | Tad1-3_Pt |
| PtHSP2 | 109735 | 109923 | C | REP-15_Pst |

Supplemental Table 1 continued.

|  | Position in sequence | |  |  |
| --- | --- | --- | --- | --- |
| BAC | Begin | End | Strand | Matching repeat |
| PtHSP4 | 3901 | 3928 | + | (CAACC)n |
| PtHSP4 | 5201 | 5369 | C | DNA7-2_Pt |
| PtHSP4 | 5370 | 5395 | C | (CGTAG)n |
| PtHSP4 | 5396 | 5667 | C | DNA7-2_Pt |
| PtHSP4 | 7055 | 7259 | C | Gypsy-5_Pgt-I |
| PtHSP4 | 10373 | 10394 | + | AT_rich |
| PtHSP4 | 10669 | 10772 | + | DNA-25_Pst |
| PtHSP4 | 13602 | 13627 | + | (G)n |
| PtHSP4 | 16639 | 16667 | C | AT_rich |
| PtHSP4 | 21716 | 21764 | + | MuDR-1_Pgt |
| PtHSP4 | 25011 | 25207 | C | DNA-25_Pst |
| PtHSP4 | 35343 | 35398 | C | (CCCCAG)n |
| PtHSP4 | 37166 | 37370 | C | Gypsy-12_Pgt-I |
| PtHSP4 | 37483 | 37563 | C | Gypsy-12_Pgt-LTR |
| PtHSP4 | 37908 | 38081 | C | REP-5_Pst |
| PtHSP4 | 38120 | 38208 | + | (TGG)n |
| PtHSP4 | 39248 | 39463 | C | DNA3-9_Pst |
| PtHSP4 | 43890 | 43917 | + | (A)n |
| PtHSP4 | 53786 | 53907 | C | REP-1_Pst |
| PtHSP4 | 53819 | 53919 | C | DNA3-2_Pt |
| PtHSP4 | 53980 | 54136 | C | Gypsy-12_Pgt-I |
| PtHSP4 | 54070 | 54146 | C | REP-5_Pst |
| PtHSP4 | 54276 | 54402 | C | Gypsy-12_Pgt-LTR |
| PtHSP4 | 55446 | 55561 | C | DNA3-2_Pt |
| PtHSP4 | 56590 | 56662 | + | EnSpm-2_Pgt |
| PtHSP4 | 57193 | 57213 | C | (A)n |
| PtHSP4 | 57267 | 57301 | C | EnSpm-2_Pgt |
| PtHSP4 | 57852 | 57920 | + | (A)n |
| PtHSP4 | 58413 | 58458 | + | (CAAAA)n |
| PtHSP4 | 59385 | 59420 | C | (TA)n |
| PtHSP4 | 59468 | 59519 | + | Tad1-2_Pt |
| PtHSP4 | 66186 | 66593 | C | DNA5-1_Pst |
| PtHSP4 | 68279 | 69042 | C | Copia-11_Pt-I |
| PtHSP4 | 69271 | 69472 | C | Harbinger-N1_Pst |
| PtHSP4 | 69645 | 69776 | + | Harbinger-N1_Pst |
| PtHSP4 | 69676 | 69790 | + | Harbinger-N1_Pst |
| PtHSP4 | 69713 | 69818 | + | Harbinger-N1_Pst |
|  | Position in sequence | |  |  |
| BAC | Begin | End | Strand | Matching repeat |
| PtHSP4 | 70005 | 70110 | C | Harbinger-N1_Pst |
| PtHSP4 | 70044 | 70326 | + | Harbinger-N1_Pst |
| PtHSP4 | 70177 | 70432 | + | Harbinger-N1_Pst |
| PtHSP4 | 71122 | 71276 | C | Copia-11_Pt-LTR |
| PtHSP4 | 71971 | 72094 | + | REP-16_Pst |
| PtHSP4 | 72619 | 72720 | + | REP-16_Pst |
| PtHSP4 | 73054 | 73188 | + | REP-16_Pst |
| PtHSP4 | 73414 | 73451 | + | AT_rich |
| PtHSP4 | 74083 | 74171 | C | REP-8_Pst |
| PtHSP4 | 74334 | 76793 | C | Gypsy-7_Pst-I |
| PtHSP4 | 77119 | 77154 | + | DNA7-1_Pt |
| PtHSP4 | 78102 | 78176 | + | DNA8-1_Pgt |
| PtHSP4 | 78117 | 78226 | + | hAT-5_Pst |
| PtHSP4 | 78456 | 78587 | C | Gypsy-15_Pgt-LTR |
| PtHSP4 | 78782 | 78860 | C | DNA3-2_Pt |
| PtHSP4 | 79743 | 79827 | + | DNA7-3_Pst |
| PtHSP4 | 83083 | 83300 | C | DNA-30_Pst |
| PtHSP4 | 83489 | 83574 | C | DNA7-3_Pst |
| PtHSP4 | 85224 | 85344 | C | LTR-1_Pt-LTR |
| PtHSP4 | 85476 | 85597 | C | LTR-1_Pt-LTR |
| PtHSP4 | 85605 | 86299 | C | hAT-5_Pst |
| PtHSP4 | 86319 | 86500 | C | MarinerN-1_Pt |
| PtHSP4 | 86530 | 88295 | C | hAT-5_Pst |
| PtHSP4 | 89185 | 89296 | C | DNA-13_Pst |
| PtHSP4 | 89400 | 89513 | + | Harbinger-10_Pst |
| PtHSP4 | 89474 | 89571 | + | REP-1_Pst |
| PtHSP4 | 89490 | 89669 | + | Harbinger-N1_Pst |
| PtHSP4 | 90354 | 90417 | + | EnSpm-2_Pgt |
| PtHSP4 | 91382 | 91464 | C | EnSpm-2_Pgt |
| PtHSP4 | 95899 | 95920 | C | (G)n |
| PtHSP4 | 99195 | 99468 | + | DNA-26_Pst |
| PtHSP4 | 99780 | 99909 | C | DNA3-6_Pst |
| PtHSP4 | 103455 | 103559 | C | (CGA)n |
| PtHSP4 | 109836 | 110010 | C | Harbinger-N1_Pt |
| PtHSP4 | 111314 | 111348 | + | (GAAA)n |
| PtHSP4 | 111458 | 113627 | C | Copia-18_Pgt-I |
| PtHSP4 | 113202 | 113975 | C | Copia-6_Pst-I |
| PtHSP4 | 114135 | 114285 | + | DNA3-2_Pt |
| PtHSP4 | 114340 | 114480 | C | (CGGGGG)n |
| PtHSP4 | 115094 | 115222 | C | Copia-6_Pst-I |
| PtHSP4 | 117476 | 117630 | C | Harbinger-N1_Pst |
|  | Position in sequence | |  |  |
| BAC | Begin | End | Strand | Matching repeat |
| PtHSP4 | 117514 | 117685 | C | Harbinger-N1_Pst |
| PtHSP4 | 120388 | 120442 | + | Gypsy-69_MLP-I |
| PtHSP4 | 121066 | 121172 | + | LTR-1_Pt-LTR |
| PtHSP4 | 121275 | 121395 | + | LTR-1_Pt-LTR |
| PtHSP4 | 122980 | 123030 | C | (TGAG)n |
| PtHSP4 | 126924 | 127101 | + | (TAG)n |
| PtHSP4 | 127104 | 127190 | + | (TAG)n |
| PtHSP4 | 130125 | 130151 | C | (TAAA)n |
| PtHSP4 | 133155 | 133363 | + | DNA7-2_Pgt |
| PtHSP4 | 133646 | 133803 | + | DNA7-2_Pgt |
| PtHSP4 | 133855 | 134000 | + | Gypsy-12_Pgt-LTR |
| PtHSP4 | 134377 | 134509 | + | Gypsy-12_Pgt-I |
| PtHSP4 | 134694 | 134730 | + | (CAAAA)n |
| PtHSP4 | 135340 | 135399 | + | (CAATA)n |
| PtHSP4 | 138918 | 138971 | + | DNA-29_Pst |
| PtHSP4 | 139869 | 139895 | C | (TAAA)n |
| PtHSP4 | 142075 | 142358 | + | DNA8-1_Pgt |
| PtHSP4 | 142238 | 142377 | + | DNA8-1_Pgt |
| PtHSP4 | 143414 | 143562 | + | Gypsy-12_Pgt-I |
| PtHSP4 | 143562 | 143723 | + | Gypsy-4_Pt-I |
| PtHSP4 | 144410 | 144473 | C | Copia-5_Pst-LTR |
| PtHSP4 | 146193 | 146268 | C | DNA3-1_Pst |
| PtHSP4 | 146285 | 146360 | C | Harbinger-10_Pst |
| PtHSP4 | 146498 | 146572 | C | Harbinger-10_Pst |
| PtHSP4 | 146545 | 146612 | C | Harbinger-N1_Pst |
| PtHSP4 | 146606 | 146703 | C | REP-1_Pst |
| PtHSP4 | 153797 | 154145 | C | Harbinger-1_Pgt |
| PtHSP4 | 157130 | 157155 | C | (CA)n |
| PtHSP4 | 161735 | 161763 | + | AT_rich |
| PtHSP4 | 164424 | 166301 | C | Helitron-1_Pgt |

Supplemental Table 2. Description of matching repeats, type of element and which rust fungus they are from.

| Matching element | Element Type | Fungal Genome |
| --- | --- | --- |
| Copia-1_Pgt-I | Copia | *P. graminis tritici* |
| Copia-1_Pgt-LTR | Copia | *P. graminis tritici* |
| Copia-1_Pst-I | Copia | *P. striiformis tritici* |
| Copia-1_Pst-LTR | Copia | *P. striiformis tritici* |
| Copia-10_Pgt-I | Copia | *P. graminis tritici* |
| Copia-10_Pgt-LTR | Copia | *P. graminis tritici* |
| Copia-10_Pt-I | Copia | *P. triticina* |
| Copia-10_Pt-LTR | Copia | *P. triticina* |
| Copia-11_Pgt-I | Copia | *P. graminis tritici* |
| Copia-11_Pgt-LTR | Copia | *P. graminis tritici* |
| Copia-11_Pt-I | Copia | *P. triticina* |
| Copia-11_Pt-LTR | Copia | *P. triticina* |
| Copia-12_Pgt-I | Copia | *P. graminis tritici* |
| Copia-12_Pgt-LTR | Copia | *P. graminis tritici* |
| Copia-13_Pgt-I | Copia | *P. graminis tritici* |
| Copia-13_Pgt-LTR | Copia | *P. graminis tritici* |
| Copia-14_Pgt-I | Copia | *P. graminis tritici* |
| Copia-14_Pgt-LTR | Copia | *P. graminis tritici* |
| Copia-15_Pgt-I | Copia | *P. graminis tritici* |
| Copia-15_Pgt-LTR | Copia | *P. graminis tritici* |
| Copia-16_Pgt-I | Copia | *P. graminis tritici* |
| Copia-16_Pgt-LTR | Copia | *P. graminis tritici* |
| Copia-17_Pgt-I | Copia | *P. graminis tritici* |
| Copia-17_Pgt-LTR | Copia | *P. graminis tritici* |
| Copia-18_Pgt-I | Copia | *P. graminis tritici* |
| Copia-18_Pgt-LTR | Copia | *P. graminis tritici* |
| Copia-2_Pgt-I | Copia | *P. graminis tritici* |
| Copia-2_Pgt-LTR | Copia | *P. graminis tritici* |
| Copia-2_Pst-I | Copia | *P. striiformis tritici* |
| Copia-2_Pst-LTR | Copia | *P. striiformis tritici* |
| Copia-3_Pgt-I | Copia | *P. graminis tritici* |
| Copia-3_Pgt-LTR | Copia | *P. graminis tritici* |
| Copia-3_Pst-I | Copia | *P. striiformis tritici* |
| Copia-3_Pst-LTR | Copia | *P. striiformis tritici* |
| Copia-4_Pgt-I | Copia | *P. graminis tritici* |
| Copia-4_Pgt-LTR | Copia | *P. graminis tritici* |
| Copia-4_Pst-I | Copia | *P. striiformis tritici* |
| Copia-4_Pst-LTR | Copia | *P. striiformis tritici* |
| Matching element | Element Type | Fungal Genome |
| Copia-5_Pgt-I | Copia | *P. graminis tritici* |
| Copia-5_Pgt-LTR | Copia | *P. graminis tritici* |
| Copia-5_Pst-I | Copia | *P. striiformis tritici* |
| Copia-5_Pst-LTR | Copia | *P. striiformis tritici* |
| Copia-6_Pgt-I | Copia | *P. graminis tritici* |
| Copia-6_Pgt-LTR | Copia | *P. graminis tritici* |
| Copia-6_Pst-I | Copia | *P. striiformis tritici* |
| Copia-7_Pgt-I | Copia | *P. graminis tritici* |
| Copia-7_Pgt-LTR | Copia | *P. graminis tritici* |
| Copia-7_Pst-LTR | Copia | *P. striiformis tritici* |
| Copia-8_Pgt-I | Copia | *P. graminis tritici* |
| Copia-8_Pgt-LTR | Copia | *P. graminis tritici* |
| Copia-9_Pgt-I | Copia | *P. graminis tritici* |
| Copia-9_Pgt-LTR | Copia | *P. graminis tritici* |
| Copia-9_Pt-I | Copia | *P. triticina* |
| Copia-9_Pt-LTR | Copia | *P. triticina* |
| DIRS-1_Pgt-I | DIRS | *P. graminis tritici* |
| DIRS-1_Pgt-LTR | DIRS | *P. graminis tritici* |
| DIRS-1_Pst-I | DIRS | *P. striiformis tritici* |
| DIRS-1_Ptt | DIRS | *P. triticina* |
| DIRS-2_Pgt-I | DIRS | *P. graminis tritici* |
| DIRS-2_Pgt-LTR | DIRS | *P. graminis tritici* |
| DIRS-2_Pst-I | DIRS | *P. striiformis tritici* |
| DIRS-3_Pgt-I | DIRS | *P. graminis tritici* |
| DIRS-3_Pgt-LTR | DIRS | *P. graminis tritici* |
| DNA-1_Pst | DNA transposon | *P. striiformis tritici* |
| DNA-10_Pst | DNA transposon | *P. striiformis tritici* |
| DNA-11_Pst | DNA transposon | *P. striiformis tritici* |
| DNA-13_Pst | DNA transposon | *P. striiformis tritici* |
| DNA-14_Pst | DNA transposon | *P. striiformis tritici* |
| DNA-15_Pst | DNA transposon | *P. striiformis tritici* |
| DNA-16_Pst | DNA transposon | *P. striiformis tritici* |
| DNA-18_Pst | DNA transposon | *P. striiformis tritici* |
| DNA-2_Pst | DNA transposon | *P. striiformis tritici* |
| DNA-22_Pst | DNA transposon | *P. striiformis tritici* |
| DNA-23_Pst | DNA transposon | *P. striiformis tritici* |
| DNA-24_Pst | DNA transposon | *P. striiformis tritici* |
| DNA-25_Pst | DNA transposon | *P. striiformis tritici* |
| DNA-26_Pst | DNA transposon | *P. striiformis tritici* |
| DNA-27_Pst | DNA transposon | *P. striiformis tritici* |
| DNA-29_Pst | DNA transposon | *P. striiformis tritici* |
| DNA-3_Pst | DNA transposon | *P. striiformis tritici* |
| Matching element | Element Type | Fungal Genome |
| DNA-30_Pst | DNA transposon | *P. striiformis tritici* |
| DNA-31_Pst | DNA transposon | *P. striiformis tritici* |
| DNA-4_Pst | DNA transposon | *P. striiformis tritici* |
| DNA-5_Pst | DNA transposon | *P. striiformis tritici* |
| DNA-6_Pst | DNA transposon | *P. striiformis tritici* |
| DNA-7_Pst | DNA transposon | *P. striiformis tritici* |
| DNA-8_Pst | DNA transposon | *P. striiformis tritici* |
| DNA-9_Pst | DNA transposon | *P. striiformis tritici* |
| DNA2-1_Pst | DNA transposon | *P. striiformis tritici* |
| DNA2-1_Pt | DNA transposon | *P. triticina* |
| DNA2-2_Pst | DNA transposon | *P. striiformis tritici* |
| DNA2-2_Pt | DNA transposon | *P. triticina* |
| DNA2-3_Pst | DNA transposon | *P. striiformis tritici* |
| DNA2-4_Pst | DNA transposon | *P. striiformis tritici* |
| DNA3-1_Pgt | DNA transposon | *P. graminis tritici* |
| DNA3-1_Pst | DNA transposon | *P. striiformis tritici* |
| DNA3-10_Pst | DNA transposon | *P. striiformis tritici* |
| DNA3-11_Pst | DNA transposon | *P. striiformis tritici* |
| DNA3-13_Pst | DNA transposon | *P. striiformis tritici* |
| DNA3-14_Pst | DNA transposon | *P. striiformis tritici* |
| DNA3-15_Pst | DNA transposon | *P. striiformis tritici* |
| DNA3-16_Pst | DNA transposon | *P. striiformis tritici* |
| DNA3-17_Pst | DNA transposon | *P. striiformis tritici* |
| DNA3-18_Pst | EnSpm | *P. striiformis tritici* |
| DNA3-19_Pst | DNA transposon | *P. striiformis tritici* |
| DNA3-2_Pst | EnSpm | *P. striiformis tritici* |
| DNA3-2_Pt | DNA transposon | *P. triticina* |
| DNA3-20_Pst | DNA transposon | *P. striiformis tritici* |
| DNA3-21_Pst | DNA transposon | *P. striiformis tritici* |
| DNA3-22_Pst | DNA transposon | *P. striiformis tritici* |
| DNA3-23_Pst | DNA transposon | *P. striiformis tritici* |
| DNA3-2B_Pst | EnSpm | *P. striiformis tritici* |
| DNA3-3_Pst | EnSpm | *P. striiformis tritici* |
| DNA3-3_Pt | DNA transposon | *P. triticina* |
| DNA3-4_Pst | DNA transposon | *P. striiformis tritici* |
| DNA3-5_Pst | DNA transposon | *P. striiformis tritici* |
| DNA3-6_Pst | DNA transposon | *P. striiformis tritici* |
| DNA3-7_Pst | DNA transposon | *P. striiformis tritici* |
| DNA3-8_Pst | DNA transposon | *P. striiformis tritici* |
| DNA3-9_Pst | DNA transposon | *P. striiformis tritici* |
| DNA5-1_Pst | DNA transposon | *P. striiformis tritici* |
| DNA6-1_Pst | DNA transposon | *P. striiformis tritici* |
| Matching element | Element Type | Fungal Genome |
| DNA7-1_Pgt | DNA transposon | *P. graminis tritici* |
| DNA7-1_Pst | DNA transposon | *P. striiformis tritici* |
| DNA7-1_Pt | DNA transposon | *P. triticina* |
| DNA7-1B_Pgt | DNA transposon | *P. graminis tritici* |
| DNA7-1C_Pgt | DNA transposon | *P. graminis tritici* |
| DNA7-1D_Pgt | DNA transposon | *P. graminis tritici* |
| DNA7-1E_Pgt | DNA transposon | *P. graminis tritici* |
| DNA7-1F_Pgt | DNA transposon | *P. graminis tritici* |
| DNA7-2_Pgt | DNA transposon | *P. graminis tritici* |
| DNA7-2_Pst | DNA transposon | *P. striiformis tritici* |
| DNA7-2_Pt | DNA transposon | *P. triticina* |
| DNA7-3_Pst | DNA transposon | *P. striiformis tritici* |
| DNA7-3_Pt | DNA transposon | *P. triticina* |
| DNA7-4_Pst | DNA transposon | *P. striiformis tritici* |
| DNA7-5_Pst | DNA transposon | *P. striiformis tritici* |
| DNA8-1_Pgt | DNA transposon | *P. graminis tritici* |
| DNA9-1_Pst | DNA transposon | *P. striiformis tritici* |
| DNA9-2_Pst | DNA transposon | *P. striiformis tritici* |
| DNAX-1_Pst | DNA transposon | *P. striiformis tritici* |
| EnSpm-1_Pgt | EnSpm | *P. graminis tritici* |
| EnSpm-1_Pst | EnSpm | *P. striiformis tritici* |
| EnSpm-2_Pgt | EnSpm | *P. graminis tritici* |
| EnSpm-3_Pgt | EnSpm | *P. graminis tritici* |
| EnSpm-N1_Pst | EnSpm | *P. striiformis tritici* |
| Gypsy-1_Pgt-I | Gypsy | *P. graminis tritici* |
| Gypsy-1_Pgt-LTR | Gypsy | *P. graminis tritici* |
| Gypsy-1_Pst-I | Gypsy | *P. striiformis tritici* |
| Gypsy-1_Pst-LTR | Gypsy | *P. striiformis tritici* |
| Gypsy-1_Pt-I | Gypsy | *P. triticina* |
| Gypsy-1_Pt-LTR | Gypsy | *P. triticina* |
| Gypsy-10_Pgt-I | Gypsy | *P. graminis tritici* |
| Gypsy-10_Pgt-LTR | Gypsy | *P. graminis tritici* |
| Gypsy-10_Pst-I | Gypsy | *P. striiformis tritici* |
| Gypsy-11_Pgt-I | Gypsy | *P. graminis tritici* |
| Gypsy-11_Pgt-LTR | Gypsy | *P. graminis tritici* |
| Gypsy-12_Pgt-I | Gypsy | *P. graminis tritici* |
| Gypsy-12_Pgt-LTR | Gypsy | *P. graminis tritici* |
| Gypsy-13_Pgt-I | Gypsy | *P. graminis tritici* |
| Gypsy-13_Pgt-LTR | Gypsy | *P. graminis tritici* |
| Gypsy-14_Pgt-I | Gypsy | *P. graminis tritici* |
| Gypsy-14_Pgt-LTR | Gypsy | *P. graminis tritici* |
| Gypsy-15_Pgt-LTR | Gypsy | *P. graminis tritici* |
| Matching element | Element Type | Fungal Genome |
| Gypsy-2_Pgt-I | Gypsy | *P. graminis tritici* |
| Gypsy-2_Pgt-LTR | Gypsy | *P. graminis tritici* |
| Gypsy-2_Pst-I | Gypsy | *P. striiformis tritici* |
| Gypsy-2_Pst-LTR | Gypsy | *P. striiformis tritici* |
| Gypsy-2_Pt-I | Gypsy | *P. triticina* |
| Gypsy-2_Pt-LTR | Gypsy | *P. triticina* |
| Gypsy-3_Pgt-I | Gypsy | *P. graminis tritici* |
| Gypsy-3_Pgt-LTR | Gypsy | *P. graminis tritici* |
| Gypsy-3_Pst-I | Gypsy | *P. striiformis tritici* |
| Gypsy-3_Pt-I | Gypsy | *P. triticina* |
| Gypsy-3_Pt-LTR | Gypsy | *P. triticina* |
| Gypsy-4_Pgt-I | Gypsy | *P. graminis tritici* |
| Gypsy-4_Pgt-LTR | Gypsy | *P. graminis tritici* |
| Gypsy-4_Pst-I | Gypsy | *P. striiformis tritici* |
| Gypsy-4_Pt-I | Gypsy | *P. triticina* |
| Gypsy-5_Pgt-I | Gypsy | *P. graminis tritici* |
| Gypsy-5_Pgt-LTR | Gypsy | *P. graminis tritici* |
| Gypsy-5_Pst-LTR | Gypsy | *P. striiformis tritici* |
| Gypsy-6_Pgt-I | Gypsy | *P. graminis tritici* |
| Gypsy-6_Pgt-LTR | Gypsy | *P. graminis tritici* |
| Gypsy-6_Pst-LTR | Gypsy | *P. striiformis tritici* |
| Gypsy-7_Pgt-I | Gypsy | *P. graminis tritici* |
| Gypsy-7_Pgt-LTR | Gypsy | *P. graminis tritici* |
| Gypsy-7_Pst-I | Gypsy | *P. striiformis tritici* |
| Gypsy-8_Pgt-I | Gypsy | *P. graminis tritici* |
| Gypsy-8_Pgt-LTR | Gypsy | *P. graminis tritici* |
| Gypsy-8_Pst-I | Gypsy | *P. striiformis tritici* |
| Gypsy-9_Pgt-I | Gypsy | *P. graminis tritici* |
| Gypsy-9_Pgt-LTR | Gypsy | *P. graminis tritici* |
| Gypsy-9_Pst-I | Gypsy | *P. striiformis tritici* |
| Harbinger-1_Pgt | Harbinger | *P. graminis tritici* |
| Harbinger-1_Pst | Harbinger | *P. striiformis tritici* |
| Harbinger-10_Pgt | Harbinger | *P. graminis tritici* |
| Harbinger-10_Pst | Harbinger | *P. striiformis tritici* |
| Harbinger-11_Pgt | Harbinger | *P. graminis tritici* |
| Harbinger-12_Pgt | Harbinger | *P. graminis tritici* |
| Harbinger-13_Pgt | Harbinger | *P. graminis tritici* |
| Harbinger-14_Pgt | Harbinger | *P. graminis tritici* |
| Harbinger-15_Pgt | Harbinger | *P. graminis tritici* |
| Harbinger-16_Pgt | Harbinger | *P. graminis tritici* |
| Harbinger-17_Pgt | Harbinger | *P. graminis tritici* |
| Harbinger-18_Pgt | Harbinger | *P. graminis tritici* |
| Matching element | Element Type | Fungal Genome |
| Harbinger-19_Pgt | Harbinger | *P. graminis tritici* |
| Harbinger-2_Pst | Harbinger | *P. striiformis tritici* |
| Harbinger-3_Pgt | Harbinger | *P. graminis tritici* |
| Harbinger-3_Pst | Harbinger | *P. striiformis tritici* |
| Harbinger-3B_Pst | Harbinger | *P. striiformis tritici* |
| Harbinger-3C_Pst | Harbinger | *P. striiformis tritici* |
| Harbinger-4_Pgt | Harbinger | *P. graminis tritici* |
| Harbinger-4_Pst | Harbinger | *P. striiformis tritici* |
| Harbinger-4B_Pst | Harbinger | *P. striiformis tritici* |
| Harbinger-4C_Pst | Harbinger | *P. striiformis tritici* |
| Harbinger-4D_Pst | Harbinger | *P. striiformis tritici* |
| Harbinger-5_Pgt | Harbinger | *P. graminis tritici* |
| Harbinger-5_Pst | Harbinger | *P. striiformis tritici* |
| Harbinger-6_Pgt | Harbinger | *P. graminis tritici* |
| Harbinger-6_Pst | Harbinger | *P. striiformis tritici* |
| Harbinger-7_Pgt | Harbinger | *P. graminis tritici* |
| Harbinger-7_Pst | Harbinger | *P. striiformis tritici* |
| Harbinger-8_Pgt | Harbinger | *P. graminis tritici* |
| Harbinger-8_Pst | Harbinger | *P. striiformis tritici* |
| Harbinger-9_Pgt | Harbinger | *P. graminis tritici* |
| Harbinger-9_Pst | Harbinger | *P. striiformis tritici* |
| Harbinger-N1_Pgt | Harbinger | *P. graminis tritici* |
| Harbinger-N1_Pst | Harbinger | *P. striiformis tritici* |
| Harbinger-N1_Pt | Harbinger | *P. triticina* |
| Harbinger-N2_Pst | Harbinger | *P. striiformis tritici* |
| Harbinger-N3_Pst | Harbinger | *P. striiformis tritici* |
| hAT-1_Pgt | hAT | *P. graminis tritici* |
| hAT-1_Pst | hAT | *P. striiformis tritici* |
| hAT-2_Pgt | hAT | *P. graminis tritici* |
| hAT-2_Pst | hAT | *P. striiformis tritici* |
| hAT-3_Pgt | hAT | *P. graminis tritici* |
| hAT-3_Pst | hAT | *P. striiformis tritici* |
| hAT-4_Pgt | hAT | *P. graminis tritici* |
| hAT-4_Pst | hAT | *P. striiformis tritici* |
| hAT-5_Pgt | hAT | *P. graminis tritici* |
| hAT-5_Pst | hAT | *P. striiformis tritici* |
| hAT-6_Pgt | hAT | *P. graminis tritici* |
| hAT-N1_Pgt | hAT | *P. graminis tritici* |
| hAT-N1_Pst | hAT | *P. striiformis tritici* |
| Helitron-1_Pgt | Helitron | *P. graminis tritici* |
| Helitron-1_Pst | Helitron | *P. striiformis tritici* |
| Helitron-1_Ptt | Helitron | *P. triticina* |
| Matching element | Element Type | Fungal Genome |
| Helitron-2_Pst | Helitron | *P. striiformis tritici* |
| Helitron-2_Ptt | Helitron | *P. triticina* |
| Helitron-3_Pst | Helitron | *P. striiformis tritici* |
| Helitron-4_Pst | Helitron | *P. striiformis tritici* |
| Helitron-5_Pst | Helitron | *P. striiformis tritici* |
| Helitron-6_Pst | Helitron | *P. striiformis tritici* |
| Helitron-6B_Pst | Helitron | *P. striiformis tritici* |
| LTR-1_Pgt-I | LTR Retrotransposon | *P. graminis tritici* |
| LTR-1_Pgt-LTR | LTR Retrotransposon | *P. graminis tritici* |
| LTR-1_Pst-LTR | LTR Retrotransposon | *P. striiformis tritici* |
| LTR-1_Ptt-I | LTR Retrotransposon | *P. triticina* |
| LTR-1_Ptt-LTR | LTR Retrotransposon | *P. triticina* |
| LTR-2_Pst-I | LTR Retrotransposon | *P. striiformis tritici* |
| LTR-2_Pst-LTR | LTR Retrotransposon | *P. striiformis tritici* |
| LTR-3_Pst-LTR | LTR Retrotransposon | *P. striiformis tritici* |
| LTR-4_Pst-LTR | LTR Retrotransposon | *P. striiformis tritici* |
| Mariner-1_Pgt | Mariner/Tc1 | *P. graminis tritici* |
| Mariner-1_Pst | Mariner/Tc1 | *P. striiformis tritici* |
| Mariner-1_Ptt | Mariner/Tc1 | *P. triticina* |
| Mariner-2_Pgt | Mariner/Tc1 | *P. graminis tritici* |
| Mariner-2_Pst | Mariner/Tc1 | *P. striiformis tritici* |
| Mariner-2_Ptt | Mariner/Tc1 | *P. triticina* |
| Mariner-3_Pgt | Mariner/Tc1 | *P. graminis tritici* |
| Mariner-3_Pst | Mariner/Tc1 | *P. striiformis tritici* |
| Mariner-3_Ptt | Mariner/Tc1 | *P. triticina* |
| Mariner-4_Pgt | Mariner/Tc1 | *P. graminis tritici* |
| Mariner-4_Ptt | Mariner/Tc1 | *P. triticina* |
| Mariner-5_Pgt | Mariner/Tc1 | *P. graminis tritici* |
| Mariner-6_Pgt | Mariner/Tc1 | *P. graminis tritici* |
| Mariner-7_Pgt | Mariner/Tc1 | *P. graminis tritici* |
| Mariner-8_Pgt | Mariner/Tc1 | *P. graminis tritici* |
| Mariner-9_Pgt | Mariner/Tc1 | *P. graminis tritici* |
| Mariner-N1_Pgt | Mariner/Tc1 | *P. graminis tritici* |
| Mariner-N1_Pst | Mariner/Tc1 | *P. striiformis tritici* |
| Mariner-N2_Pgt | Mariner/Tc1 | *P. graminis tritici* |
| Mariner-N2_Pst | Mariner/Tc1 | *P. striiformis tritici* |
| Mariner-N2_Ptt | Mariner/Tc1 | *P. triticina* |
| Mariner-N2B_Pst | Mariner/Tc1 | *P. striiformis tritici* |
| Mariner-N3_Pgt | Mariner/Tc1 | *P. graminis tritici* |
| Mariner-N3_Pst | Mariner/Tc1 | *P. striiformis tritici* |
| Mariner-N3_Ptt | Mariner/Tc1 | *P. triticina* |
| Mariner-N4_Pgt | Mariner/Tc1 | *P. graminis tritici* |
| Matching element | Element Type | Fungal Genome |
| Mariner-N5_Pgt | Mariner/Tc1 | *P. graminis tritici* |
| MarinerN-1_Pt | Mariner/Tc1 | *P. triticina* |
| MuDR-1_Pgt | MuDR | *P. graminis tritici* |
| MuDR-1_Pst | MuDR | *P. striiformis tritici* |
| MuDR-2_Pgt | MuDR | *P. graminis tritici* |
| MuDR-2_Pst | MuDR | *P. striiformis tritici* |
| MuDR-3_Pgt | MuDR | *P. graminis tritici* |
| MuDR-N1_Pgt | MuDR | *P. graminis tritici* |
| MuDRF-1_Pgt | MuDR | *P. graminis tritici* |
| MuDRF-1_Pst | MuDR | *P. striiformis tritici* |
| MuDRF-1_Ptt | MuDR | *P. triticina* |
| MuDRF-1B_Pst | MuDR | *P. striiformis tritici* |
| MuDRF-2_Pgt | MuDR | *P. graminis tritici* |
| MuDRF-2_Pst | MuDR | *P. striiformis tritici* |
| MuDRF-3_Pst | MuDR | *P. striiformis tritici* |
| MuDRF-N1_Pst | MuDR | *P. striiformis tritici* |
| P-1_Pgt | P | *P. graminis tritici* |
| P-1_Pst | P | *P. striiformis tritici* |
| P-1_Ptt | P | *P. triticina* |
| P-N1_Ptt | P | *P. triticina* |
| REP-1_Pst | Repetitive element | *P. striiformis tritici* |
| REP-10_Pst | Repetitive element | *P. striiformis tritici* |
| REP-11_Pst | Repetitive element | *P. striiformis tritici* |
| REP-12_Pst | Repetitive element | *P. striiformis tritici* |
| REP-13_Pst | Repetitive element | *P. striiformis tritici* |
| REP-14_Pst | Repetitive element | *P. striiformis tritici* |
| REP-15_Pst | Repetitive element | *P. striiformis tritici* |
| REP-16_Pst | Repetitive element | *P. striiformis tritici* |
| REP-17_Pst | Repetitive element | *P. striiformis tritici* |
| REP-2_Pst | Repetitive element | *P. striiformis tritici* |
| REP-3_Pst | Repetitive element | *P. striiformis tritici* |
| REP-4_Pst | Repetitive element | *P. striiformis tritici* |
| REP-5_Pst | Repetitive element | *P. striiformis tritici* |
| REP-6_Pst | Repetitive element | *P. striiformis tritici* |
| REP-7_Pst | Repetitive element | *P. striiformis tritici* |
| REP-8_Pst | Repetitive element | *P. striiformis tritici* |
| REP-9_Pst | Repetitive element | *P. striiformis tritici* |
| Sagan-1_Pgt | Mariner/Tc1 | *P. graminis tritici* |
| Sagan-1_Pst | Mariner/Tc1 | *P. striiformis tritici* |
| Sagan-2_Pgt | Mariner/Tc1 | *P. graminis tritici* |
| Sagan-2_Pst | Mariner/Tc1 | *P. striiformis tritici* |
| Sagan-N1_Pgt | Mariner/Tc1 | *P. graminis tritici* |
| Matching element | Element Type | Fungal Genome |
| Sagan-N1_Pst | Mariner/Tc1 | *P. striiformis tritici* |
| Sagan-N2_Pgt | Mariner/Tc1 | *P. graminis tritici* |
| Sagan-N3_Pgt | Mariner/Tc1 | *P. graminis tritici* |
| SAT-1_Pgt | SAT | *P. graminis tritici* |
| Tad1-1_Pgt | Tad1 | *P. graminis tritici* |
| Tad1-1_Pst | Tad1 | *P. striiformis tritici* |
| Tad1-1_Ptt | Tad1 | *P. triticina* |
| Tad1-1B_Pgt | Tad1 | *P. graminis tritici* |
| Tad1-2_Pgt | Tad1 | *P. graminis tritici* |
| Tad1-2_Ptt | Tad1 | *P. triticina* |
| Tad1-3_Pgt | Tad1 | *P. graminis tritici* |
| Tad1-3_Ptt | Tad1 | *P. triticina* |
| Tad1-4_Pgt | Tad1 | *P. graminis tritici* |
| Tad1-4_Ptt | Tad1 | *P. triticina* |
| Tad1-5_Pgt | Tad1 | *P. graminis tritici* |
| Tad1-6_Pgt | Tad1 | *P. graminis tritici* |
